# FIN-Seq: transcriptional profiling of specific cell types from frozen archived tissue of the human central nervous system

**DOI:** 10.1093/nar/gkz968

**Published:** 2019-11-15

**Authors:** Ryoji Amamoto, Emanuela Zuccaro, Nathan C Curry, Sonia Khurana, Hsu-Hsin Chen, Constance L Cepko, Paola Arlotta

**Affiliations:** 1 Department of Stem Cell and Regenerative Biology, Harvard University, Cambridge, MA 02138, USA; 2 Department of Genetics and Ophthalmology, Howard Hughes Medical Institute, Blavatnik Institute, Harvard Medical School, Boston, MA 02115, USA; 3 Stanley Center for Psychiatric Research, Broad Institute of MIT and Harvard, Cambridge, MA 02142, USA

## Abstract

Thousands of frozen, archived tissue samples from the human central nervous system (CNS) are currently available in brain banks. As recent developments in RNA sequencing technologies are beginning to elucidate the cellular diversity present within the human CNS, it is becoming clear that an understanding of this diversity would greatly benefit from deeper transcriptional analyses. Single cell and single nucleus RNA profiling provide one avenue to decipher this heterogeneity. An alternative, complementary approach is to profile isolated, pre-defined cell types and use methods that can be applied to many archived human tissue samples that have been stored long-term. Here, we developed FIN-Seq (Frozen Immunolabeled Nuclei Sequencing), a method that accomplishes these goals. FIN-Seq uses immunohistochemical isolation of nuclei of specific cell types from frozen human tissue, followed by bulk RNA-Sequencing. We applied this method to frozen postmortem samples of human cerebral cortex and retina and were able to identify transcripts, including low abundance transcripts, in specific cell types.

## INTRODUCTION

The human central nervous system (CNS) comprises an extremely diverse set of cell types. While this heterogeneity has been appreciated since the work of early anatomists, it was not until recently that different cell types of the CNS have begun to be defined at the molecular level (1–9). Two of the most well studied CNS areas, the cerebral cortex and retina, have been the subjects of some of the earliest molecular characterizations, leading to the identification of at least 16 neuronal subtypes in the adult human cerebral cortex (4) and 18 major cell types in the adult human retina (7). While these pioneering studies have started to highlight the heterogeneity of the adult human CNS, more fine-grained distinctions among cell types are likely present. These distinctions will become more apparent with an increased number of cells profiled, and/or greater depth in sequencing of individual cell types. Such studies will greatly enable our understanding of the development and function of cell types in health and disease.

Transcriptional profiling to define cell types among heterogeneous populations, or to define gene expression features among different cell types, are now frequently carried out using single cell RNA sequencing (10–13). Although a very powerful approach, single cell RNA sequencing does not provide a depth of coverage of rare cell types, unless a very large number of cells is sequenced. An alternative is to use bulk RNA sequencing of defined, potentially rare, cell types, to avoid sequencing a large number of more abundant cell types. The discovery of novel markers has facilitated the isolation of specific cell types from diverse tissues, with isolation based on genetic markers, dyes, or antibodies (14–19).

Most postmortem human tissue is preserved by fixation or flash-freezing. While whole-cell approaches are incompatible with flash-frozen CNS tissue, the nuclei from frozen tissue stay intact and can be profiled. In addition, nuclear RNA has been successfully used as a proxy for the cellular transcriptome (4,20–24). Single nucleus RNA sequencing has been used to profile neuronal subtypes from frozen human cerebral cortex tissue (4). Bulk sequencing of immunolabeled nuclei also has been used to characterize the transcriptome of specific cell types in frozen human postmortem cerebellum (25). This example provides encouragement to explore further the use of frozen samples for antibody-based FACS purification of specific cell populations and subsequent RNA profiling.

Thousands of frozen human postmortem brain tissue samples, including those with disease, are readily available through brain banks. These samples are a valuable resource that is immediately available. A significant number of samples are archived, which, given the wide genetic variation among humans, will be important for the interpretation of disease-specific changes. This resource has not been fully exploited due to technical limitations in the retrieval of cell type specific RNA from frozen specimens. It also has been unclear whether long term storage, over a period of decades, would lead to diminished RNA quality and/or antigen detection.

Here, we developed FIN-Seq (Frozen Immunolabeled Nuclei Sequencing), a technique that combines nuclear isolation, fixation, immunolabeling, FACS, and RNA sequencing from frozen, archived human CNS tissue. While some antibodies such as those against NeuN and SOX6 are known to work with fresh tissue (26), a simple method to apply a wider range of antibodies against cell-type specific markers in archived frozen tissue has not been available until recently (25). With FIN-Seq, we isolated and profiled specific excitatory and inhibitory neuronal subtypes from frozen human cerebral cortex tissue, some of which had been stored for over fifteen years. We also extended the method to a different part of the CNS by isolating and profiling cone photoreceptors from the frozen human retina. Successful isolation of cone photoreceptors, which constitute roughly 2% of retinal cells, signified that rare populations could be reliably profiled from a frozen tissue sample. Interestingly, we also found that the nuclear transcripts captured with FIN-Seq represented more of the whole-cell transcriptome than has been seen using single nuclei sequencing (24). FIN-Seq is cost-effective and provides access to the transcriptomes of user-defined cell types from widely-available frozen human CNS samples.

## MATERIALS AND METHODS

### Mouse brain samples

All animals were handled according to protocols approved by the Institutional Animal Care and Use Committee (IACUC) of Harvard University. For each biological replicate, the neocortex of P30 or adult (1+ years old) CD1 mice were microdissected, flash-frozen in an isopentane/dry ice slurry, and stored at −80°C.

### Frozen human CNS samples

Frozen Brodmann Area 4 (Primary Motor Cortex) samples of Patient 1569, 3529, 3589, 4340 and 5650 were obtained from Human Brain and Spinal Fluid Resource Center at University of California, Los Angeles through the NIH NeuroBioBank. Patient 1569 was a 61-year-old male with no clinical brain diagnosis and the postmortem interval was 9 h. Patient 3529 was a 58-year-old male with no clinical brain diagnosis and the postmortem interval was 9 h. Patient 3589 was a 53-year-old male with no clinical brain diagnosis and the postmortem interval was 15 h. Patient 4340 was a 47-year-old male with no clinical brain diagnosis and the postmortem interval was 12.5 h. Patient 5650 was a 55-year-old male with no clinical brain diagnosis and the postmortem interval was 22.6 h. This IRB protocol (IRB16-2037) was determined to be not human subjects research by the Harvard University-Area Committee on the Use of Human Subjects.

Frozen eyes were obtained from Restore Life USA (Elizabethton, TN) through TissueForResearch. Patient DRLU032618A was a 52-year-old female with no clinical eye diagnosis and the postmortem interval was 8 h. Patient DRLU041518A was a 57-year-old male with no clinical eye diagnosis and the postmortem interval was 5 h. Patient DRLU041818C was a 53-year-old female with no clinical eye diagnosis and the postmortem interval was 9 h. Patient DRLU051918A was a 43-year-old female with no clinical eye diagnosis and the postmortem interval was 5 h. This IRB protocol (IRB17-1781) was determined to be not human subjects research by the Harvard University-Area Committee on the Use of Human Subjects.

### Nuclei Isolation, Immunolabeling, and FACS

Thawed tissue was minced and immediately incubated in 1% PFA (with 1 μl ml^−1^ RNasin Plus (Promega, Madison, WI)) for 5 min. Nuclei were prepared by Dounce homogenizing in 0.1% Triton X-100 homogenization buffer (250 mM sucrose, 25 mM KCl, 5 mM MgCl_2_, 10 mM Tris buffer, pH 8.0, 1 μM DTT, 1× Protease Inhibitor (Promega), Hoechst 33342 10 ng ml^−1^ (Thermo Fisher Scientific, Waltham, MA, USA), 0.1% Triton X-100, 1 μl ml^−1^ RNasin Plus). Sample was then overlaid on top of 20% sucrose bed (25 mM KCl, 5 mM MgCl_2_, 10 mM Tris buffer, pH 8.0) and spun at 500×g for 12 min at 4°C. The pellet was resuspended in 4% PFA (with 1 ul ml^−1^ RNasin Plus) and incubated for 15 min on ice. The sample was spun at 2000×g for 5 min at 4°C and the supernatant was discarded. The sample was then resuspended in blocking buffer (0.5% BSA in nuclease-free PBS, 0.5 μl ml^−1^ RNasin Plus) and incubated for 15 min. Sample was spun and the pellet was resuspended and incubated in primary antibody (1:50 SATB2 antibody (Abcam, Cambridge, UK), 1:100 BCL11B antibody (Abcam), 1:1,000 CAR antibody (kind gift from Dr Sheryl Craft) in blocking buffer) for 30 min at 4°C. After washing 1× with blocking buffer, the sample was incubated in secondary antibody (1:750 appropriate AlexaFluor secondary antibodies (Thermo Fisher Scientific)) for 30 min at 4°C. After 1× wash, the sample was passed through a 35μm filter (Corning, Corning, NY, USA) before proceeding to FACS using FACSAria (BD Biosciences, Franklin Lakes, NJ, USA). 2N nuclei were determined by a Hoechst histogram. From the 2N nuclei, those that ran along the diagonal were considered to be the negative population. This was evident from the secondary antibody-only control FACS plot (Supplementary Figure S1), where single nuclei events displayed a continuum of autofluorescence in a diagonal line with events showing high fluorescence in one laser channel (e.g. 647nm) that were also were high in another laser channel (e.g. 594 nm). Nuclei that were highly fluorescent in all laser channels were thus also considered to be negative. Traditional gating methods with four quadrants include the events that display high fluorescence in all channels. Using the diagonal line to assess the negative cells then allowed the selection of the positive population, i.e. those nuclei that were right-shifted or left-shifted compared to the nuclei on the diagonal. Isolated populations were sorted into blocking buffer. Sorted nuclei were spun at 3000×g for 7 min, and the supernatant was discarded. A step-by-step online protocol is available at https://www.protocols.io/view/fin-seq-frozen-immunolabeled-nuclei-sequencing-zxbf7in.

### RNA isolation and library preparation

RNA was extracted using the RecoverAll Total Nuclear Isolation Kit (Thermo Fisher Scientific). Crosslinking was reversed by incubating the nuclear pellet in Digestion Buffer and Protease mixture (100 μl buffer and 4 μl protease) for 3 h at 50°C, which differs from the manufacturer's protocol. The downstream steps were according to the manufacturer's protocol. RNA-seq libraries were generated using the SMART-Seq v.4 Ultra Low Input RNA kit (Takara Bio, Kusatsu, Japan) and Nextera XT DNA Library Prep Kit (Illumina, San Diego, CA, USA) according to the manufacturer's protocol. The number of cycles was determined based on the number of nuclei sorted, as indicated in the SMART-Seq v.4 protocol. However, given the reduced amount of RNA in nuclei, at least 2 cycles were added from the protocol recommendation. The Nextera XT Kit uses 150 pg total cDNA as the input after SMART-Seq v.4; therefore, SMART-Seq v.4 should generate in the range of 1–2 ng of cDNA. The cDNA library fragment size was determined by the BioAnalyzer 2100 HS DNA Assay (Agilent, Santa Clara, CA, USA). The libraries were sequenced as paired-end reads on HiSeq 2500 or NextSeq 500.

### RNA-seq data processing

Quality control of RNA-seq reads were performed using fastqc version 0.11.5 (https://www.bioinformatics.babraham.ac.uk/projects/fastqc/). RNA-seq reads were clipped and mapped onto the mouse genome (Ensembl GRCm38.88) or human genome (Ensembl GRCh38.87) using STAR version 2.5 (27,28). Parameters used were as follows: –runThreadN 6 –readFilesCommand zcat –outSAMtype BAM SortedByCoordinate –outSAMunmapped Within –outSAMattributes Standard –clip3pAdapterSeq –quantMode TranscriptomeSAM GeneCounts

Alignment quality control was performed using Qualimap version 2.2.1 (29). Read counts were generated by HT-seq version 0.6.1 (30). Sample parameters used were as follows: -i gene_name -s no.

The resulting matrix of read counts were analyzed for differential expression by DESeq2 version 3.5 (31). Samples with non-neuronal cell contamination were discarded for analysis (BCL11B^+^ 3529 and BCL11B^+^ 3589). For the DE analysis of human retina samples, any genes with more than five samples with zero reads were discarded. The R scripts used for differential expression analysis are available in Supplementary Files.

### Gene set enrichment analysis

GSEAPreranked analysis was performed on the All versus SATB2^+^ dataset using GSEA v3.0 (32). Gene set databases including markers that define neuronal subtypes identified by Darmanis *et al.* (2) and Lake *et al.* (4) were generated. Parameters used were as follows: Number of permutations: 1000; Enrichment statistic: classic; the ranked file was generated using log2FoldChange generated by DESeq2. To determine significance, we used the default FDR <0.25 for all gene sets.

### RNAscope

P30 and adult (>1 year old) mouse brains were perfused with 4% PFA in PBS and sectioned on a cryostat at a thickness of 16 μm. Double *in situ* fluorescence hybridization was performed using the RNAscope Fluorescent Multiplex assay according to the manufacturer's protocol (Advanced Cell Diagnostics, Newark, CA, USA). The following probes were used for the mouse study: Satb2-C1 (Catalog #: 413261), Satb2-C2 (Catalog#: 420981-C2), Bcl11b-C3 (Catalog#: 413271-C3), Ddit4l-C1 (Catalog#: 468551), Kcnn2-C1 (Catalog#: 427971), Unc5d-C2 (Catalog#: 480461-C2) and Rprm-C2 (Catalog#: 466071-C2).

FFPE adult human cerebral cortex tissue from a 54-year-old female was obtained from Abcam (ab4296). Chromogenic double *in situ* hybridization was performed for the human brain tissue using the RNAscope 2.5 HD Duplex Assay (Advanced Cell Diagnostics) according to the manufacturer's protocol. A Fluorescent Multiplex assay was used for the human retina tissue according to the manufacturer's protocol (Advanced Cell Diagnostics). The following probes were used for the human study: SATB2-C2 (Catalog#: 420981-C2), RORB-C1 (Catalog#: 446061), UNC5D-C1 (Catalog#: 459991), CRYM-C2 (Catalog#: 460031-C2), GAD1-C1 (Catalog#: 404031), COL6A1-C1 (Catalog#: 482461), ANXA1-C1 (Catalog#: 465411), ARR3-C2 (Catalog#: 486461-C2), DHRS3-C1 (Catalog#: 504941), RAB41-C1 (Catalog#: 559561).

### Immunohistochemistry

P30 and adult (>1 year old) mouse brains were perfused with 4% PFA in PBS and sectioned on a vibratome at a thickness of 40 μm. Immunohistochemistry was performed as previously described (14) with anti-SATB2 and anti-BCL11B antibodies.

FFPE adult human cerebral cortex tissue from a 54-year-old female was obtained from Abcam (ab4296). The brain tissue was deparaffinized by 2× xylene incubation (3 min each) followed by 1 × 100% ethanol (3 min), 1 × 95% ethanol (3 min), 1 × 70% ethanol (3 min) washes. Antigen retrieval was performed in a citrate buffer (10 mM citric acid, pH 6.0) in a rice cooker with boiling water for 20 min. Subsequently, immunohistochemistry was performed as described above with an additional step of incubation in TrueBlack (Biotium, Fremont, CA, USA) after incubation in blocking buffer to quench the lipofuscin autofluorescence.

For human eye immunohistochemistry, formalin-fixed human postmortem eyes were obtained from Restore Life USA. Patient DRLU101818C was a 54-year-old male with no clinical eye diagnosis and the postmortem interval was 4 h. Patient DRLU110118A was a 59-year-old female with no clinical eye diagnosis and the postmortem interval was 4 h. The retina was cryosectioned at 16 μm thickness. Immunohistochemistry was performed as previously described (14) with anti-CAR antibody (1:10,000).

### Imaging

Fluorescent confocal images of the brain were acquired with Zeiss LSM 700 confocal microscope and analyzed with the ZEN Black software. Fluorescent confocal images of the retina were acquired with Nikon Ti inverted spinning disk microscope and analyzed with the NIS-Elements software. Brightfield color images of the human brain were acquired with AxioZoom V16 Zoom Microscope.

## RESULTS

### FACS isolation of immunolabeled nuclei from frozen mouse brain samples

**Figure 1.**
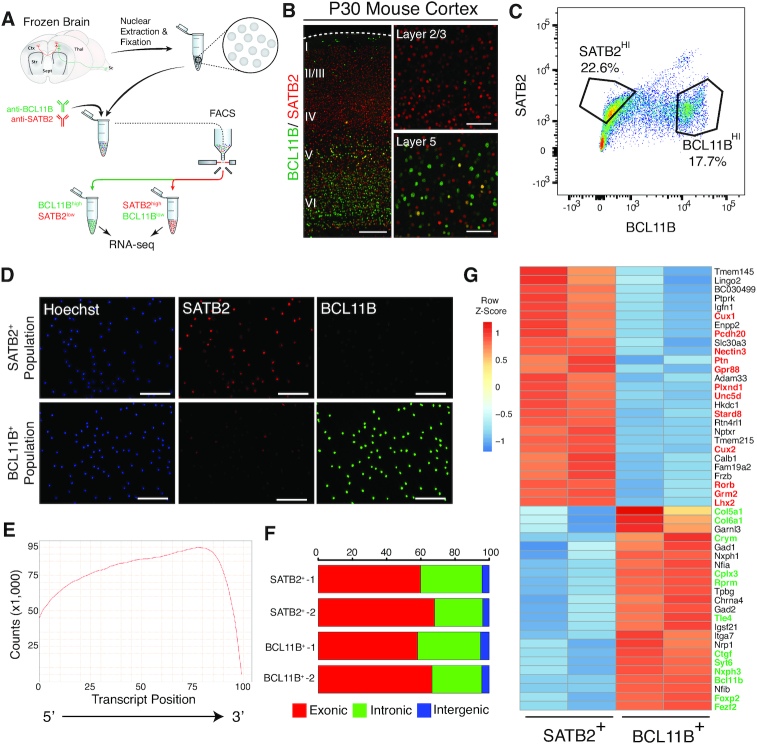
Isolation and transcriptome sequencing of two neuronal subtypes from the frozen mouse neocortex. (**A**) Schematic of FIN-Seq for frozen adult mouse brain. Nuclei were extracted from the frozen mouse neocortex by Dounce homogenization. The nuclei were fixed and immunolabeled with anti-BCL11B and anti-SATB2 antibodies. Two nuclear populations were isolated by FACS based on expression level of these two proteins. The nuclei were reverse crosslinked by protease digestion, and the RNA was extracted. Sequencing libraries were generated and subsequently sequenced to obtain cell type specific transcriptomes. (**B**) Representative immunohistochemistry images using BCL11B and SATB2 antibodies in the P30 mouse neocortex showed SATB2 expression in the upper layers and BCL11B expression in the deep layers (Left image). In layer 5, there were sparse cells that express both SATB2 and BCL11B (bottom right image). (**C**) FACS plot of nuclei labeled with SATB2 and BCL11B antibodies showed a cluster of nuclei immunolabeled with BCL11B and a cluster of nuclei labeled with SATB2. (**D**) Isolated nuclei were counterstained by the Hoechst dye and either SATB2 or BCL11B in the SATB2^+^ population (top panels) or BCL11B^+^ population (bottom panels). (**E**) Representative quantification of read counts mapped by transcript position (5′ to 3′) for every gene. (**F**) Representative quantification of percentage of read counts mapped to exonic, intronic, or intergenic regions of the genome. (**G**) Heatmap of unbiased top 50 differentially expressed genes between SATB2^+^ and BCL11B^+^ populations. Known markers of callosal projection neurons (in red) were enriched in the SATB2^+^ population while known markers of corticofugal projection neurons (in green) were enriched in the BCL11B^+^ population. Scale bars; 100 μm (B, right panels, D), 500 μm (B, left panel).

To test whether sequencing of nuclear RNA from frozen tissue is feasible, specific nuclear populations from frozen mouse neocortex were isolated. To this end, modifications were made to protocols that use intracellular antibody staining to isolate specific cell types (17,33–36). Since these protocols were for intact cells, which cannot be dissociated from frozen tissue, we developed a protocol for the isolation of antibody-labeled nuclei (Figure 1A). Isolation of nuclei eliminates the need for enzymatic dissociation, which induces aberrant activation of immediate early genes (37). We added a 1% PFA step before the extraction of the nuclei to ensure the structural integrity of the nuclei. In addition, our method of RNA extraction using the modified Recoverall Kit protocol, which includes a three-hour protease step, outperformed other FFPE RNA extraction kits in terms of RNA recovery.

From the flash-frozen neocortex of P30 mice, we sought to isolate two populations of projection neurons, Corticofugal Projection Neurons (CFuPN) and Callosal Projection Neurons (CPN). In the adult mouse brain, BCL11B (also known as CTIP2) is largely expressed in CFuPNs in layer 5b and 6 and in sparse populations of interneurons. SATB2 is expressed by CPNs in all layers (38) (Figure 1B). BCL11B and SATB2 expression are largely mutually exclusive, with a small population of layer 5 neurons expressing both markers (17,39) (Figure 1B, layer 5, inset). Upon isolation by homogenization, nuclei were fixed, immunolabeled with antibodies against BCL11B and SATB2, and separated into two populations by FACS: SATB2^LO^BCL11B^HI^ (BCL11B^+^) and SATB2^HI^BCL11B^LO^ (SATB2^+^) (*n* = 2 for each population) (Figure 1C, D, Supplementary Figure S1). On average, we collected 55,215 BCL11B^+^ nuclei and 102,016 SATB2^+^ nuclei per biological replicate. These results indicate that this protocol could result in the isolation of intact nuclei that are immunolabeled with specific intranuclear antibodies.

To determine whether the correct neuronal populations were isolated, and to test nuclear transcriptional profiling using these samples, SMART-Seq v.4 RNA-seq libraries were generated and sequenced on HiSeq 2500. For each sample, libraries were sequenced to a mean of 40 million 100 bp paired-end reads (range: 36–48 million reads per sample) to be able to reliably detect low-abundance transcripts. To determine the degree of RNA degradation, we measured the 3′ bias using Qualimap (29). The 3′ bias for P30 samples ranged from 0.65 to 0.69 (mean±SD: 0.685±0.02), which is comparable to RNA Integrity Number (RIN) of 2–4 (40) (Figure 1E). Consistent with the idea that nuclear transcripts are predominantly nascent RNA, we found that a substantial number of reads mapped to intronic regions (Exonic: 63.16±4.89%; Intronic: 32.49±4.48%; Intergenic: 4.36±0.56%) (Figure 1F) (4,22,24). Distribution of normalized read counts was virtually identical among samples (Supplementary Figure S2A). Unbiased hierarchical clustering showed that the samples of the same population clustered together (average Pearson correlation between samples within population: *r* = 0.98) (Supplementary Figure S2B). Subsequently, the two populations were analyzed for differential (gene) expression (DE). The frequency distribution of all *P-*values showed an even distribution of null *P-*values, thus allowing for calculation of adjusted *P-*value using the Benjamini-Hochberg procedure (Supplementary Figure S2C). Between populations, we found 2,698 differentially expressed genes (adjusted *P-*value < 0.05) out of 17,662 genes (Supplementary Figure S2D). The high number of genes detected suggests identification of low abundance transcripts.

From the DE analysis, we found an enrichment of known CPN and Layer 4 (L4) markers (e.g. *Cux2, Unc5d* and *Rorb*) in the SATB2^+^ population among the unbiased top 50 DE genes. Conversely, we found an enrichment of CFuPN markers (e.g. *Fezf2, Foxp2*, and *Crym*) in the BCL11B^+^ population (Figure 1G). BCL11B also labels interneurons in all layers of the mouse neocortex (14,41). Accordingly, we found an enrichment of some interneuron markers in the BCL11B^+^ population (e.g. *Gad1* and *Gad2*) (Figure 1G). To confirm the molecular identities of the isolated neuronal populations, we also determined the relative expression levels of known CPN and CFuPN marker genes that were differentially expressed between CPN and CFuPN in previous studies (21 CPN markers and 22 CFuPN markers) (14,17,38). We found that all CPN markers were enriched in the SATB2^+^ population and all CFuPN markers were enriched in the BCL11B^+^ population (Supplementary Figure S3). To validate the differentially expressed genes, we chose four DE genes (*Ddit4l, Unc5d, Kcnn2* and *Rprm*) for further analysis. Using RNAscope double fluorescent *in situ* hybridization (FISH), we localized the transcripts of these genes in specific neuronal populations. We found that *Ddit4l* and *Unc5d* were expressed in layers 2 through 4 and were localized to *Satb2^+^* neurons (Supplementary Figure S4). Additionally, *Kcnn2* and *Rprm* were expressed in layers 5 and 6, respectively, and they were specifically confined to *Bcl11b^+^* neurons (Supplementary Figure S4). In addition, we successfully isolated and profiled the same neuronal populations from mature, adult (1+ years old) mouse neocortex (Supplementary Figures S5 and S6). These results indicate that FIN-Seq can be used to isolate CFuPN and CPN nuclei from flash-frozen mouse neocortex for downstream quantitative RNA-seq analysis of specific neuronal populations.

To determine the degree to which nuclear transcript abundance correlates to cellular transcript abundance, we sought to compare the transcriptional profiles of BCL11B^+^ nuclei and cells. For cells, we dissociated the brains of P7 mice using a protocol described previously (17). For nuclei, we performed the FIN-Seq protocol, starting with a fresh P7 brain instead of flash-freezing to keep the starting material consistent between cells and nuclei. We chose the P7 time point because dissociation of the adult mouse brain into single cells affects cell viability at later ages (42,43). Transcriptional analysis of BCL11B^+^ cells and nuclei showed a high degree of correlation (average Pearson correlation between cellular versus nuclear: *r* = 0.90; cellular versus cellular: *r* = 0.93; nuclear versus nuclear: *r* = 0.93) (Supplementary Figure S7). In contrast, previous comparison of single nucleus and single cell transcriptomes from the adult mouse brain showed a lower degree of correlation (*r* = 0.77) (24). These results indicate that bulk sequencing of isolated nuclei using FIN-Seq could more accurately represent the transcript abundance found within whole cells.

### Specific neuronal subtypes can be isolated from frozen postmortem human brain samples

**Figure 2.**
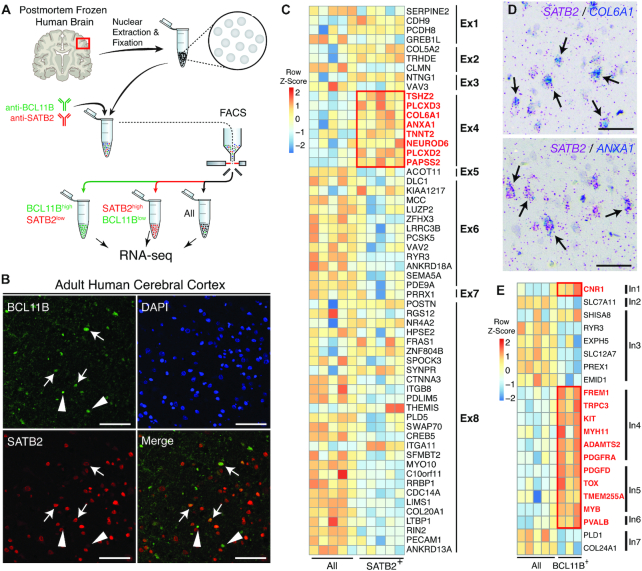
Isolation and profiling of neuronal subtypes from the frozen human cerebral cortex. (**A**) Schematic of FIN-Seq for frozen human cerebral cortex. Nuclei were isolated and subsequently fixed in 4% PFA. They were immunolabeled with anti-BCL11B and anti-SATB2 antibodies, and FACS isolated into populations. RNA from the nuclei were sequenced to obtain a cell type specific transcriptome. (**B**) Representative immunohistochemistry of the adult human cerebral cortex using anti-BCL11B and anti-SATB2 antibodies. Some nuclei expressed both SATB2 and BCL11B (arrows), some nuclei expressed BCL11B but not SATB2 (arrowheads), and many nuclei expressed SATB2 but not BCL11B. (**C**) A heatmap representing relative expression levels of excitatory neuron markers previously identified by single nuclei RNA sequencing that are differentially expressed (adjusted *P-*value < 0.05) between SATB2^+^ and All populations. Markers of neuronal subtype Ex4 (outlined in red), which expresses *SATB2*, were enriched in the SATB2^+^ population. (**D**) Validation of Ex4 markers, *COL6A1* (left panel) and *ANXA1* (right panel) using RNAscope single molecule FISH. Both *COL6A1* and *ANXA1* were expressed in *SATB2*^+^ neurons (arrows). (**E**) A heatmap representing relative expression levels of inhibitory neuron markers previously identified by single nuclei RNA sequencing that are differentially expressed (adjusted *P-*value < 0.05) between BCL11B^+^ and All populations. Markers of neuronal subtypes, In1, In4, In5 and In6, all of which express *BCL11B*, were enriched in the BCL11B^+^ population. Scale bars: 100 μm (B), 50 μm (D).

To determine whether this protocol is applicable to frozen postmortem samples of the human brain, we obtained five frozen postmortem brain samples (Brodmann area 4, primary motor cortex; ages: 47–61) from a tissue bank that had stored them long-term (for description of the samples, see Materials and Methods). Of note, the oldest frozen sample had been archived for over 25 years. We implemented the same FIN-Seq protocol as above to the frozen human cortical tissue (Figure 2A), in which we found BCL11B^+^/SATB2^−^, BCL11B^+^/SATB2^+^ and BCL11B^−^/SATB2^+^ nuclei (Figure 2B). Although the precise identity of these neurons remains unclear due to the lack of data on additional features, including their electrophysiological properties and patterns of connectivity, the transcriptomic data indicate that they are subtypes of excitatory and inhibitory neurons (4). We used FACS to isolate SATB2^LO^BCL11B^HI^ and SATB2^HI^BCL11B^LO^ nuclei as well as all cortical nuclei (henceforth called BCL11B^+^, SATB2^+^ and all, respectively) for comparison (BCL11B^+^: 26,616 nuclei/replicate, *n* = 5; SATB2^+^: 104,865 nuclei/replicate, *n* = 5; All: 67,580 nuclei/replicate, *n* = 5) (Supplementary Figure S8). These results indicate that nuclear isolation of specific neuronal subtypes from frozen postmortem human brain tissue is feasible using this technique.

To determine the molecular identity of the isolated neuronal populations, we performed RNA sequencing of each population (BCL11B^+^, SATB2^+^, and All, sequenced to a mean of 36 million paired-end 100 bp reads). The average RIN of the frozen human brain samples prior to FIN-Seq was 3.9. After FIN-Seq, the 3′ bias ranged from 0.69 to 0.78 (mean ± SD: 0.73 ± 0.02), which corresponds to a RIN of 2–4, indicating that the FIN-Seq protocol does not further decrease the integrity of the RNA (Supplementary Figure S9A). The human brain contains an increased number of nascent transcripts compared to other organs and organisms (44). Accordingly, we found that the proportion of intronic reads was higher in the human neuronal samples compared to that in mice (exonic: 47.76 ± 5.82%; intronic: 45.51 ± 5.04%; intergenic: 6.72 ± 1.13%) (Supplementary Figure S9B). Quality control of the sequencing reads and differential expression analysis indicated successful sample separation and differential expression analysis (Supplementary Figure S10). Interestingly, we did not observe an increase in 3′ sequencing bias with an increase in the number of years that the frozen specimens had been stored at −80°C (Supplementary Figure S10). Between SATB2^+^ and All populations, we found 4,917 differentially expressed genes (adjusted *P-*value < 0.05) out of 24,979 genes. Between BCL11B^+^ and All populations, we found 2,812 differentially expressed genes (adjusted *P-*value < 0.05) out of 24,477 genes (Supplementary Figure S10).

To determine the molecular identity of the SATB2^+^ and BCL11B^+^ populations, we first compared the gene expression levels of known markers of oligodendrocytes, astrocytes, and neurons. We found that neuronal markers were enriched in both SATB2^+^ and BCL11B^+^ populations. However, two of the BCL11B^+^ samples were discarded following this analysis due to enrichment of oligodendrocyte and astrocyte markers, indicating non-neuronal cell contamination. We also found an enrichment in the BCL11B^+^ population of *PDGFRA*, normally considered an oligodendrocyte marker, but also previously shown to be expressed by a subset of inhibitory neurons in the human cerebral cortex (Supplementary Figure S11) (4). The SATB2^+^ population highly expressed *SLC17A7* (also known as *VGLUT1*) and did not express *GAD1* or *GAD2*, while the BCL11B^+^ population expressed *GAD1* and *GAD2* at high levels, indicating that, while SATB2^+^ population contained mainly excitatory neurons, the BCL11B^+^ population contained also inhibitory neurons (Supplementary Figure S12).

We next sought to understand the identity of the SATB2^+^ and BCL11B^+^ populations at the neuronal subtype-level. Previously, single nucleus RNA-seq has identified eight excitatory neuronal subtypes (Ex1–Ex8) and eight inhibitory neuronal subtypes (In1–In8) in the adult human neocortex (4). *SATB2* is expressed in all excitatory neurons, but it is most highly expressed in one of the neuronal subtypes referred to, in this prior study, as Ex4. *BCL11B* is highly expressed in In1, In4, In5, and In6. *SATB2* and *BCL11B* are both expressed in Ex6 and Ex8, but we would not expect to see these subtypes in our populations as we did not collect the SATB2^HI^BCL11B^HI^ population. For the SATB2^+^ population, we cross-referenced our DE gene set (adjusted *P-*value < 0.05) to the molecular signature genes that define the eight excitatory cortical neuronal subtypes (Ex1–Ex8). From this analysis, we observed a high level of expression of Ex4 markers in the SATB2^+^ population compared to the All population (Figure 2E). To confirm these results, we also ran the dataset through a gene set enrichment analysis (GSEA) against all marker genes that define Ex1–Ex8 (32). We found that Ex4 gene set was significantly enriched in the SATB2^+^ population while Ex6 and Ex8 gene sets were enriched in the All population (default significance at FDR < 0.25; Ex4: FDR = 0.139; Ex6: FDR = 0.043; Ex8: FDR = 0.005). Depletion of Ex6 and Ex8 from the SATB2^+^ population is likely due to the exclusion of SATB2^HI^BCL11B^HI^ nuclei. We confirmed the expression of *COL6A1* and *ANXA1*, two Ex4 markers, in *SATB2*^+^ neurons by single molecule FISH (Figure 2D). In the BCL11B^+^ population, we found that the markers for In1, In4, In5 and In6 were enriched compared to the All population (Figure 2E). To directly compare the BCL11B^+^ population and the SATB2^+^ population, we cross-referenced our DE gene set (adjusted *P-*value < 0.05) to the molecular signature genes that define the eight excitatory and eight inhibitory cortical neuronal subtypes (Ex1–Ex8 and In1–In8). From this analysis, we saw that the majority of Ex4 markers were enriched in the SATB2^+^ population while Ex6, In4 and In5 markers were enriched in the BCL11B^+^ population (Supplementary Figure S13). Furthermore, previous single cell sequencing of the fresh adult human brain identified seven neuronal communities (NC), of which *SATB2* is highly expressed in neuronal community 4 (NC4) (2). Accordingly, we found that the markers for NC4 are highly expressed in the isolated SATB2^+^ population (Supplementary Figure S14). By GSEA analysis, we also found that NC4 gene set was significantly enriched in the SATB2^+^ population (FDR = 0.037). Taken together, our results show the FIN-Seq protocol can isolate neuronal subtypes defined by antibody staining for downstream transcriptional profiling from frozen postmortem human cortical samples.

### Isolation and transcriptional profiling of cone photoreceptors from the human retina

**Figure 3.**
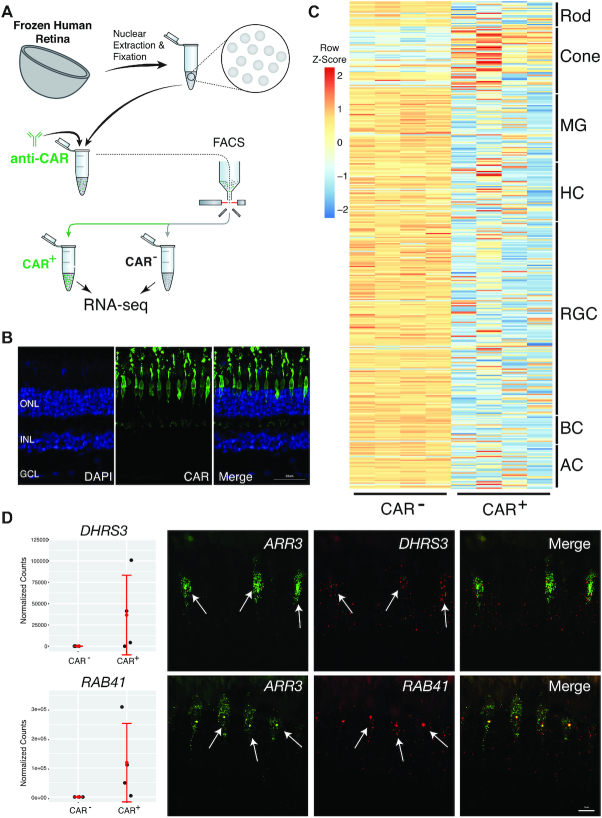
Isolation and sequencing of cone photoreceptor nuclei from the frozen human retina. (**A**) Schematic of FIN-Seq for the frozen human retina. Nuclei were extracted from the frozen retina and subsequently fixed in 4% PFA. Nuclei were then immunolabeled with an anti-CAR antibody and sorted. CAR^+^ and CAR^−^ populations were obtained and the nuclear RNA was sequenced. (**B**) Representative immunohistochemistry of an adult human retina section using the anti-CAR antibody (middle panel) and DAPI (left panel). CAR^+^ cone photoreceptors were localized to the uppermost layer of the ONL. (**C**) A heatmap representing relative expression levels of retinal cell type markers previously identified by single nuclei RNA sequencing (adjusted *P-*value < 1E−10) that are differentially expressed (adjusted *P-*value < 0.05) between CAR^−^ and CAR^+^ populations. Cone markers were enriched in the CAR^+^ population. (**D**) Validation of new human cone photoreceptor markers by single molecule FISH. Expression levels for *DHRS3* and *RAB41* from the RNA-seq are indicated in the graphs (left panels). Both *DHRS3* and *RAB41* were expressed in the *ARR3*^+^ cone photoreceptors (arrows). ONL, Outer Nuclear Layer; INL, Inner Nuclear Layer; GCL, Ganglion Cell Layer; MG, Müller Glia; AC, Amacrine Cell; HC, Horizontal Cell; RGC, Retinal Ganglion Cell; BC, Bipolar Cell. Scale bars; 50 μm (B), 10 μm (D).

To determine whether we could use FIN-Seq to isolate and profile specific cell types from another region of the human CNS, we chose to isolate cone photoreceptors from the retina. Cones comprise only ∼2–4% of the human retina (6,7) so this was also a test of the ability of FIN-Seq to allow for a deeper transcriptomic analysis of a relatively rare cell type. We obtained four freshly frozen postmortem eyes (age range: 40–60, see Materials and Methods for description of samples) from patients without known retinal disorders. Nuclei were extracted from the mid-peripheral retina, fixed, and immunostained by a human Cone Arrestin (CAR, also known as ARR3) antibody (Figure 3A). In human retinal cross-sections, we found CAR expression in the nuclei and cell bodies of cone photoreceptors, located in the outer nuclear layer where all photoreceptors reside (Figure 3B). CAR^+^ and CAR^−^ nuclei were isolated by FACS, and the RNA was extracted for deep sequencing (CAR^+^: 8,500 nuclei/replicate, *n* = 4; CAR^−^: 180,000 nuclei/replicate, *n* = 4). On average, 1.97% of all nuclei were CAR^+^, a proportion similar to known percentage of cone photoreceptors in the human retina based on single cell RNA sequencing (6,7). To determine whether fixation was necessary for antibody penetration, we performed the FIN-Seq protocol with and without fixation. We found that the distinct CAR^+^ population was present only with fixation, suggesting that, unlike the NeuN antibody, fixation is necessary for optimal immunolabeling of CAR (Supplementary Figure S15). cDNA sequencing libraries were generated using SMART-Seq v.4 and sequenced to a mean depth of 43 million (range: 37–53 million reads/replicate) 75 bp paired-end reads. The sequencing reads were analyzed, and the quality control parameters indicated successful sample separation and differential expression analysis (Supplementary Figure S16). We found 5,260 DE genes (adjusted *P-*value < 0.05) out of 12,910 genes between CAR^+^ and CAR^−^ nuclear populations.

To determine the cellular identity of the CAR^+^ population, we examined the top 50 differential expressed genes between CAR^+^ and CAR^−^ populations. Of the 16 genes enriched in the CAR^+^ population in the top 50 DE genes, eight are known markers of human cone photoreceptors, identified by previous single cell RNA sequencing experiments (Supplementary Figure S17, cone markers in red) (7). We then cross-referenced our DE gene set (adjusted *P-*value < 0.05) to the retinal cell type specific markers identified by single nucleus RNA sequencing (adjusted *P-*value < 1E−10) (6). We found that cone specific markers were upregulated in the CAR^+^ population while other cell-type specific markers were enriched in the CAR^−^ population (Figure 3C). We also performed single molecule FISH for two previously uncharacterized cone markers, *RAB41* and *DHRS3*, and found that they were specifically expressed in *ARR3^+^* cone photoreceptors (Figure 3D). These results indicate that FIN-Seq enabled successful isolation and transcriptional profilng of cone photoreceptors from frozen postmortem human retinas.

## DISCUSSION

Technologies for the profiling of RNA expression within the CNS are rapidly expanding. At the tissue level, analyses of gene expression within distinct regions of the fetal and adult human brain have been carried out (45–56). Despite progress, these tissue-level approaches cannot account for the cellular heterogeneity of the brain, an organ with tremendous cellular diversity. This is important especially when human CNS disorders are under study, as histological studies have underscored the cell type-specific nature of cellular dysfunction and degeneration (57–59). Insights into the mechanism of such diseases may result from the isolation and transcriptional profiling of the specific neuronal populations affected. These may be rare cell types within the tissue, further underscoring the need for technologies that allow enrichment of defined cell types.

Recent developments of single cell RNA-seq technology have enabled unbiased sampling of all cell types from a human CNS tissue sample (1–9). However, for some types of studies, it is impractical to assess the gene expression changes in all cell types. If the cell type of interest is known, bulk RNA-seq of isolated neuronal populations is a complementary approach to quantify gene expression more comprehensively in specific cells of interest. Here, we developed a method, FIN-Seq, to quantify gene expression in isolated neuronal populations from frozen archived postmortem human CNS tissue. Bulk RNA sequencing can lead to the detection of low abundance transcripts and rare splice variants, which are often not detected in single cell or single nucleus RNA sequencing (60,61). We also show that bulk nuclear sequencing can represent more of the transcriptome of the entire cell, compared to single nucleus sequencing. Moreover, as suggested by the data from cone photoreceptors, which comprise only 2% of retinal cells, FIN-seq may prove to be especially valuable for the deep profiling of rare cell types.

The challenge of applying FIN-Seq for some cell types is the availability of suitable nuclear antibodies. With the rapid progress of single cell sequencing, markers of molecularly distinct human neuronal subtypes are becoming available. For most of these markers, however, no antibody exists. FIN-Seq could greatly benefit from efforts to generate a validated antibody catalog such as the Protein Capture Reagents Program, in which over 700 validated monoclonal antibodies against human transcription factors have been produced (62). Interestingly, ER proteins also have been used to purify cell type-specific human nuclei using FACS, which could significantly increase the number of available cell type-specific antibodies (25). For molecular markers without an antibody, FIN-Seq could be further developed to isolate specific cell populations using nuclear RNA by FISH techniques such as RNAscope or SABER (63,64). Labeling specific nuclear transcripts of human neuronal nuclei for downstream FACS and transcriptome sequencing will enable FIN-Seq to capture any cell type of interest.

Taken together, FIN-Seq can enable transcriptional profiling of specific neuronal subtypes in the postmortem human CNS, without a need for genetic labeling. Counting only those from the NIH brain bank, over 16,000 postmortem samples are available, including those with neurological disorders, and many of them are stored long-term as flash-frozen samples. With FIN-Seq, we can start to interrogate the transcriptional changes that accompany specific neuronal subtypes in the adult human brain and identify molecular mechanisms underlying cell type specific pathology.

## DATA AVAILABILITY

The mouse transcriptome data have been deposited to GEO (GEO Accession # GSE130143).

## Supplementary Material

gkz968_Supplemental_Files

## Notes

Present address: Emanuela Zuccaro, Department of Biomedical Sciences, University of Padova, 35131 Padova, Italy.

## References

[1] Cherry T.J., Yang M.G., Harmin D.A., Tao P., Timms A.E., Bauwens M., Allikmets R., Jones E.M., Chen R., DeBaere E. et al. Epigenomic profiling and single-nucleus-RNA-seq reveal cis-regulatory elements in human retina, macula and rpe and non-coding genetic variation. 2018; bioRxiv doi:08 September 2018, preprint: not peer reviewed 10.1101/412361.

[2] Darmanis S., Sloan S.A., Zhang Y., Enge M., Caneda C., Shuer L.M., Hayden Gephart M.G., Barres B.A., Quake S.R. A survey of human brain transcriptome diversity at the single cell level. Proc. Natl. Acad. Sci. U.S.A. 2015; 112:7285–7290. 26060301 10.1073/pnas.1507125112 PMC4466750

[3] Hodge R.D., Bakken T.E., Miller J.A., Smith K.A., Barkan E.R., Graybuck L.T., Close J.L., Long B., Penn O., Yao Z. et al. Conserved cell types with divergent features between human and mouse cortex. 2018; bioRxiv doi:05 August 2018, preprint: not peer reviewed 10.1101/384826. PMC6919571 31435019

[4] Lake B.B., Ai R., Kaeser G.E., Salathia N.S., Yung Y.C., Liu R., Wildberg A., Gao D., Fung H.-L.L., Chen S. et al. Neuronal subtypes and diversity revealed by single-nucleus RNA sequencing of the human brain. Science (New York, N.Y.). 2016; 352:1586–1590. 10.1126/science.aaf1204 PMC5038589 27339989

[5] Lake B.B., Chen S., Sos B.C., Fan J., Kaeser G.E., Yung Y.C., Duong T.E., Gao D., Chun J., Kharchenko P.V. et al. Integrative single-cell analysis of transcriptional and epigenetic states in the human adult brain. Nat. Biotechnol. 2018; 36:70–80. 29227469 10.1038/nbt.4038 PMC5951394

[6] Liang Q., Dharmat R., Owen L., Shakoor A., Li Y., Kim S., Vitale A., Kim I., Morgan D., Wu N. et al. Single-nuclei RNA-seq on human retinal tissue provides improved transcriptome profiling. 2019; bioRxiv doi:11 November 2018, preprint: not peer reviewed 10.1101/468207. PMC6917696 31848347

[7] Lukowski S., Lo C., Sharov A., Nguyen Q., Fang L., Hung S., Zhu L., Zhang T., Nguyen T., Senabouth A. et al. Generation of human neural retina transcriptome atlas by single cell RNA sequencing. 2018; bioRxiv doi:24 September 2018, preprint: not peer reviewed 10.1101/425223.

[8] Peng Y.-R.R., Shekhar K., Yan W., Herrmann D., Sappington A., Bryman G.S., van Zyl T., Do M.T.H.T.H., Regev A., Sanes J.R. Molecular classification and comparative taxonomics of foveal and peripheral cells in primate retina. Cell. 2019; 176:1222. 30712875 10.1016/j.cell.2019.01.004 PMC6424338

[9] Phillips M.J., Jiang P., Howden S., Barney P., Min J., York N.W., Chu L.-F.F., Capowski E.E., Cash A., Jain S. et al. A novel approach to single cell rna-sequence analysis facilitates in silico gene reporting of human pluripotent stem cell-derived retinal cell types. Stem Cells. 2018; 36:313–324. 29230913 10.1002/stem.2755 PMC5823737

[10] Macosko E.Z., Basu A., Satija R., Nemesh J., Shekhar K., Goldman M., Tirosh I., Bialas A.R., Kamitaki N., Martersteck E.M. et al. Highly parallel genome-wide expression profiling of individual cells using nanoliter droplets. Cell. 2015; 161:1202–1214. 26000488 10.1016/j.cell.2015.05.002 PMC4481139

[11] Shekhar K., Lapan S.W., Whitney I.E., Tran N.M., Macosko E.Z., Kowalczyk M., Adiconis X., Levin J.Z., Nemesh J., Goldman M. et al. Comprehensive classification of retinal bipolar neurons by single-cell transcriptomics. Cell. 2016; 166:1308. 27565351 10.1016/j.cell.2016.07.054 PMC5003425

[12] Tasic B., Menon V., Nguyen T.N., Kim T.K., Jarsky T., Yao Z., Levi B., Gray L.T., Sorensen S.A., Dolbeare T. et al. Adult mouse cortical cell taxonomy revealed by single cell transcriptomics. Nat. Neurosci. 2016; 19:335–346. 26727548 10.1038/nn.4216 PMC4985242

[13] Zeisel A., Muñoz-Manchado A.B., Codeluppi S., Lönnerberg P., La Manno G., Juréus A., Marques S., Munguba H., He L., Betsholtz C. et al. Brain structure. Cell types in the mouse cortex and hippocampus revealed by single-cell RNA-seq. Science (New York, N.Y.). 2015; 347:1138–1142. 10.1126/science.aaa1934 25700174

[14] Arlotta P., Molyneaux B.J., Chen J., Inoue J., Kominami R., Macklis J.D. Neuronal subtype-specific genes that control corticospinal motor neuron development in vivo. Neuron. 2005; 45:207–221. 15664173 10.1016/j.neuron.2004.12.036

[15] Heiman M., Schaefer A., Gong S., Peterson J.D., Day M., Ramsey K.E., Suárez-Fariñas M., Schwarz C., Stephan D.A., Surmeier D.J. et al. A translational profiling approach for the molecular characterization of CNS cell types. Cell. 2008; 135:738–748. 19013281 10.1016/j.cell.2008.10.028 PMC2696821

[16] Lobo M.K., Karsten S.L., Gray M., Geschwind D.H., Yang X.W. FACS-array profiling of striatal projection neuron subtypes in juvenile and adult mouse brains. Nat. Neurosci. 2006; 9:443–452. 16491081 10.1038/nn1654

[17] Molyneaux B.J., Goff L.A., Brettler A.C., Chen H.-H.H., Brown J.R., Hrvatin S., Rinn J.L., Arlotta P. DeCoN: genome-wide analysis of in vivo transcriptional dynamics during pyramidal neuron fate selection in neocortex. Neuron. 2015; 85:275–288. 25556833 10.1016/j.neuron.2014.12.024 PMC4430475

[18] Siegert S., Cabuy E., Scherf B.G., Kohler H., Panda S., Le Y.-Z.Z., Fehling H.J., Gaidatzis D., Stadler M.B., Roska B. Transcriptional code and disease map for adult retinal cell types. Nat. Neurosci. 2012; 15:487. 22267162 10.1038/nn.3032

[19] Telley L., Govindan S., Prados J., Stevant I., Nef S., Dermitzakis E., Dayer A., Jabaudon D. Sequential transcriptional waves direct the differentiation of newborn neurons in the mouse neocortex. Science (New York, N.Y.). 2016; 351:1443–1446. 10.1126/science.aad8361 26940868

[20] Barthelson R.A., Lambert G.M., Vanier C., Lynch R.M., Galbraith D.W. Comparison of the contributions of the nuclear and cytoplasmic compartments to global gene expression in human cells. BMC Genomics. 2007; 8:340. 17894886 10.1186/1471-2164-8-340 PMC2048942

[21] Grindberg R.V., Yee-Greenbaum J.L., McConnell M.J., Novotny M., O'Shaughnessy A.L., Lambert G.M., Araúzo-Bravo M.J., Lee J., Fishman M., Robbins G.E. et al. RNA-sequencing from single nuclei. Proc. Natl. Acad. Sci. U.S.A. 2013; 110:19802–19807. 24248345 10.1073/pnas.1319700110 PMC3856806

[22] Habib N., Avraham-Davidi I., Basu A., Burks T., Shekhar K., Hofree M., Choudhury S.R., Aguet F., Gelfand E., Ardlie K. et al. Massively parallel single-nucleus RNA-seq with DroNc-seq. Nat. methods. 2017; 14:955–958. 28846088 10.1038/nmeth.4407 PMC5623139

[23] Krishnaswami S.R., Grindberg R.V., Novotny M., Venepally P., Lacar B., Bhutani K., Linker S.B., Pham S., Erwin J.A., Miller J.A. et al. Using single nuclei for RNA-seq to capture the transcriptome of postmortem neurons. Nat. Protoc. 2016; 11:499–524. 26890679 10.1038/nprot.2016.015 PMC4941947

[24] Lake B.B., Codeluppi S., Yung Y.C., Gao D., Chun J., Kharchenko P.V., Linnarsson S., Zhang K. A comparative strategy for single-nucleus and single-cell transcriptomes confirms accuracy in predicted cell-type expression from nuclear RNA. Sci. Rep. 2017; 7:6031. 28729663 10.1038/s41598-017-04426-w PMC5519641

[25] Xu X., Stoyanova E.I., Lemiesz A.E., Xing J., Mash D.C., Heintz N. Species and cell-type properties of classically defined human and rodent neurons and glia. eLife. 2018; 7:e37551. 30320555 10.7554/eLife.37551 PMC6188473

[26] Kozlenkov A., Li J., Apontes P., Hurd Y.L., Byne W.M., Koonin E.V., Wegner M., Mukamel E.A., Dracheva S. A unique role for DNA (hydroxy)methylation in epigenetic regulation of human inhibitory neurons. Sci. Adv. 2018; 4:eaau6190. 30263963 10.1126/sciadv.aau6190 PMC6157969

[27] Aken B.L., Achuthan P., Akanni W., Amode M.R., Bernsdorff F., Bhai J., Billis K., Carvalho-Silva D., Cummins C., Clapham P. et al. Ensembl 2017. Nucleic Acids Res. 2017; 45:D635–D642. 27899575 10.1093/nar/gkw1104 PMC5210575

[28] Dobin A., Davis C.A., Schlesinger F., Drenkow J., Zaleski C., Jha S., Batut P., Chaisson M., Gingeras T.R. STAR: ultrafast universal RNA-seq aligner. Bioinformatics. 2013; 29:15–21. 23104886 10.1093/bioinformatics/bts635 PMC3530905

[29] Okonechnikov K., Conesa A., García-Alcalde F. Qualimap 2: advanced multi-sample quality control for high-throughput sequencing data. Bioinformatics. 2016; 32:292–294. 26428292 10.1093/bioinformatics/btv566 PMC4708105

[30] Anders S., Pyl P.T., Huber W. HTSeq–a Python framework to work with high-throughput sequencing data. Bioinformatics. 2015; 31:166–169. 25260700 10.1093/bioinformatics/btu638 PMC4287950

[31] Love M.I., Huber W., Anders S. Moderated estimation of fold change and dispersion for RNA-seq data with DESeq2. Genome Biol. 2014; 15:550. 25516281 10.1186/s13059-014-0550-8 PMC4302049

[32] Subramanian A., Tamayo P., Mootha V.K., Mukherjee S., Ebert B.L., Gillette M.A., Paulovich A., Pomeroy S.L., Golub T.R., Lander E.S. et al. Gene set enrichment analysis: a knowledge-based approach for interpreting genome-wide expression profiles. Proc. Natl. Acad. Sci. U.S.A. 2005; 102:15545–15550. 16199517 10.1073/pnas.0506580102 PMC1239896

[33] Hrvatin S., Deng F., O’Donnell C.W., Gifford D.K., Melton D.A. MARIS: method for analyzing RNA following intracellular sorting. PLoS One. 2014; 9:e89459. 24594682 10.1371/journal.pone.0089459 PMC3940959

[34] Pechhold S., Stouffer M., Walker G., Martel R., Seligmann B., Hang Y., Stein R., Harlan D.M., Pechhold K. Transcriptional analysis of intracytoplasmically stained, FACS-purified cells by high-throughput, quantitative nuclease protection. Nat. Biotechnol. 2009; 27:1038–1042. 19838197 10.1038/nbt.1579 PMC4638177

[35] Pan Y., Ouyang Z., Wong W.H., Baker J.C. A new FACS approach isolates hESC derived endoderm using transcription factors. PLoS One. 2011; 6:e17536. 21408072 10.1371/journal.pone.0017536 PMC3052315

[36] Yamada H., Maruo R., Watanabe M., Hidaka Y., Iwatani Y., Takano T. Messenger RNA quantification after fluorescence activated cell sorting using intracellular antigens. Biochem. Biophys. Res. Commun. 2010; 397:425–428. 20510885 10.1016/j.bbrc.2010.05.112

[37] Lacar B., Linker S.B., Jaeger B.N., Krishnaswami S.R., Barron J.J., Kelder M.J.E.J.E., Parylak S.L., Paquola A.C.M.C.M., Venepally P., Novotny M. et al. Nuclear RNA-seq of single neurons reveals molecular signatures of activation. Nat. Commun. 2016; 7:11022. 27090946 10.1038/ncomms11022 PMC4838832

[38] Molyneaux B.J., Arlotta P., Menezes J.R., Macklis J.D. Neuronal subtype specification in the cerebral cortex. Nat. Rev. Neurosci. 2007; 8:427–437. 17514196 10.1038/nrn2151

[39] Harb K., Magrinelli E., Nicolas C.S.S., Lukianets N., Frangeul L., Pietri M., Sun T., Sandoz G., Grammont F., Jabaudon D. et al. Area-specific development of distinct projection neuron subclasses is regulated by postnatal epigenetic modifications. eLife. 2016; 5:e09531. 26814051 10.7554/eLife.09531 PMC4744182

[40] Sigurgeirsson B., Emanuelsson O., Lundeberg J. Sequencing degraded RNA addressed by 3′ tag counting. PLoS One. 2014; 9:e91851. 24632678 10.1371/journal.pone.0091851 PMC3954844

[41] Nikouei K., Muñoz-Manchado A.B., Hjerling-Leffler J. BCL11B/CTIP2 is highly expressed in GABAergic interneurons of the mouse somatosensory cortex. J. Chem. Neuroanat. 2016; 71:1–5. 26698402 10.1016/j.jchemneu.2015.12.004

[42] Habib N., Li Y., Heidenreich M., Swiech L., Avraham-Davidi I., Trombetta J.J., Hession C., Zhang F., Regev A. Div-Seq: Single-nucleus RNA-Seq reveals dynamics of rare adult newborn neurons. Science (New York, N.Y.). 2016; 353:925–928. 10.1126/science.aad7038 PMC5480621 27471252

[43] Saxena A., Wagatsuma A., Noro Y., Kuji T., Asaka-Oba A., Watahiki A., Gurnot C., Fagiolini M., Hensch T.K., Carninci P. Trehalose-enhanced isolation of neuronal sub-types from adult mouse brain. BioTechniques. 2012; 52:381–385. 22668417 10.2144/0000113878 PMC3696583

[44] Ameur A., Zaghlool A., Halvardson J., Wetterbom A., Gyllensten U., Cavelier L., Feuk L. Total RNA sequencing reveals nascent transcription and widespread co-transcriptional splicing in the human brain. Nat. Struct. Mol. Biol. 2011; 18:1435–1440. 22056773 10.1038/nsmb.2143

[45] Hawrylycz M.J., Lein E.S., Guillozet-Bongaarts A.L., Shen E.H., Ng L., Miller J.A., van de Lagemaat L.N., Smith K.A., Ebbert A., Riley Z.L. et al. An anatomically comprehensive atlas of the adult human brain transcriptome. Nature. 2012; 489:391–399. 22996553 10.1038/nature11405 PMC4243026

[46] Kang H.J., Kawasawa Y.I., Cheng F., Zhu Y., Xu X., Li M., Sousa A.M.M.M., Pletikos M., Meyer K.A., Sedmak G. et al. Spatio-temporal transcriptome of the human brain. Nature. 2011; 478:483–489. 22031440 10.1038/nature10523 PMC3566780

[47] Wang X.-S.S., Simmons Z., Liu W., Boyer P.J., Connor J.R. Differential expression of genes in amyotrophic lateral sclerosis revealed by profiling the post mortem cortex. Amyotroph. Lateral Scler. 2006; 7:201–210. 17127558 10.1080/17482960600947689

[48] Lederer C.W., Torrisi A., Pantelidou M., Santama N., Cavallaro S. Pathways and genes differentially expressed in the motor cortex of patients with sporadic amyotrophic lateral sclerosis. BMC Genomics. 2007; 8:26. 17244347 10.1186/1471-2164-8-26 PMC1796866

[49] Dangond F., Hwang D., Camelo S., Pasinelli P., Frosch M.P., Stephanopoulos G., Stephanopoulos G., Brown R.H., Gullans S.R. Molecular signature of late-stage human ALS revealed by expression profiling of postmortem spinal cord gray matter. Physiol. Genomics. 2004; 16:229–239. 14645737 10.1152/physiolgenomics.00087.2001

[50] Offen D., Barhum Y., Melamed E., Embacher N., Schindler C., Ransmayr G. Spinal cord mRNA profile in patients with ALS: comparison with transgenic mice expressing the human SOD-1 mutant. J. Mol. Neurosci. 2009; 38:85–93. 18651250 10.1007/s12031-007-9004-z

[51] Bossers K., Meerhoff G., Balesar R., van Dongen J.W., Kruse C.G., Swaab D.F., Verhaagen J. Analysis of gene expression in Parkinson's disease: possible involvement of neurotrophic support and axon guidance in dopaminergic cell death. Brain Pathol. 2009; 19:91–107. 18462474 10.1111/j.1750-3639.2008.00171.x PMC8094761

[52] Miller R.M., Kiser G.L., Kaysser-Kranich T.M., Lockner R.J., Palaniappan C., Federoff H.J. Robust dysregulation of gene expression in substantia nigra and striatum in Parkinson's disease. Neurobiol. Dis. 2006; 21:305–313. 16143538 10.1016/j.nbd.2005.07.010

[53] Hauser M.A., Li Y.-J.J., Xu H., Noureddine M.A., Shao Y.S., Gullans S.R., Scherzer C.R., Jensen R.V., McLaurin A.C., Gibson J.R. et al. Expression profiling of substantia nigra in Parkinson disease, progressive supranuclear palsy, and frontotemporal dementia with parkinsonism. Arch. Neurol. 2005; 62:917–921. 15956162 10.1001/archneur.62.6.917

[54] Moran L.B., Duke D.C., Deprez M., Dexter D.T., Pearce R.K., Graeber M.B. Whole genome expression profiling of the medial and lateral substantia nigra in Parkinson's disease. Neurogenetics. 2006; 7:1–11. 16344956 10.1007/s10048-005-0020-2

[55] Papapetropoulos S., Ffrench-Mullen J., McCorquodale D., Qin Y., Pablo J., Mash D.C. Multiregional gene expression profiling identifies MRPS6 as a possible candidate gene for Parkinson's disease. Gene Expr. 2006; 13:205–215. 17193926 10.3727/000000006783991827 PMC6032441

[56] Dumitriu A., Latourelle J.C., Hadzi T.C., Pankratz N., Garza D., Miller J.P., Vance J.M., Foroud T., Beach T.G., Myers R.H. Gene expression profiles in Parkinson disease prefrontal cortex implicate FOXO1 and genes under its transcriptional regulation. PLos Genet. 2012; 8:e1002794. 22761592 10.1371/journal.pgen.1002794 PMC3386245

[57] Hartong D.T., Berson E.L., Dryja T.P. Retinitis pigmentosa. Lancet (London, England). 2006; 368:1795–1809. 10.1016/S0140-6736(06)69740-7 17113430

[58] Mitchell J.D., Borasio G.D. Amyotrophic lateral sclerosis. Lancet (London, England). 2007; 369:2031–2041. 10.1016/S0140-6736(07)60944-1 17574095

[59] Sulzer D., Surmeier D.J. Neuronal vulnerability, pathogenesis, and Parkinson's disease. Mov. Disord. 2013; 28:715–724. 23589357 10.1002/mds.25187

[60] Arzalluz-Luque Á., Conesa A. Single-cell RNAseq for the study of isoforms-how is that possible. Genome Biol. 2018; 19:110. 30097058 10.1186/s13059-018-1496-z PMC6085759

[61] Liu S., Trapnell C. Single-cell transcriptome sequencing: recent advances and remaining challenges [version 1; peer review: 2 approved]. F1000Research. 2016; 5:182. 10.12688/f1000research.7223.1 PMC4758375 26949524

[62] Venkataraman A., Yang K., Irizarry J., Mackiewicz M., Mita P., Kuang Z., Xue L., Ghosh D., Liu S., Ramos P. et al. A toolbox of immunoprecipitation-grade monoclonal antibodies to human transcription factors. Nat. Methods. 2018; 15:330–338. 29638227 10.1038/nmeth.4632 PMC6063793

[63] Kishi J.Y., Lapan S.W., Beliveau B.J., West E.R., Zhu A., Sasaki H.M., Saka S.K., Wang Y., Cepko C.L., Yin P. SABER amplifies FISH: enhanced multiplexed imaging of RNA and DNA in cells and tissues. Nat. Methods. 2019; 16:533–544. 31110282 10.1038/s41592-019-0404-0 PMC6544483

[64] Klemm S., Semrau S., Wiebrands K., Mooijman D., Faddah D.A., Jaenisch R., van Oudenaarden A. Transcriptional profiling of cells sorted by RNA abundance. Nat. Methods. 2014; 11:549–551. 24681693 10.1038/nmeth.2910 PMC4174458

