# Supplementary material for: FIN-Seq: transcriptional profiling of specific cell types from frozen archived tissue of the human central nervous system
Source: Nucleic Acids Res. 2019 Nov 15;48(1):e4. doi: 10.1093/nar/gkz968 (PMC7145626; doi:10.1093/nar/gkz968)
Supplement: gkz968_Supplemental_Files [file gkz968_supplemental_files.zip › Supplementary Figures.pdf]

Supplementary Figures

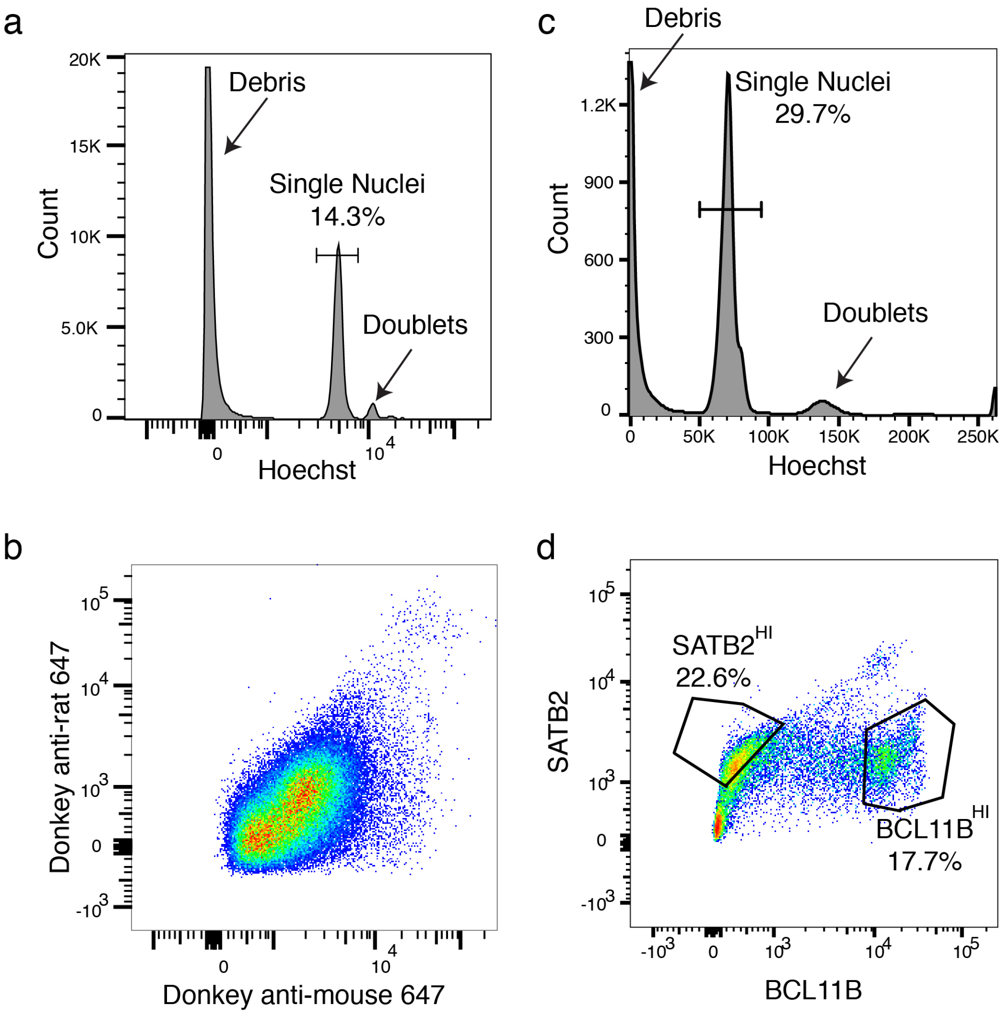

Supplementary Figure 1: FACS plot for obtaining SATB2^{HI} and BCL11B^{HI} populations.

a) All events are first plotted as a histogram of Hoechst intensity (405 nm laser). The peak near 0 is the debris that does not contain DNA. The second peak corresponds to single nuclei (14.3% of all events). The peak to the right corresponds to doublets. b) FACS plot of secondary antibody lasers (647 nm on y-axis, 594 nm on x-axis) used for the secondary-only control sample. The control nuclei are displayed in a diagonal line. c) Hoechst histogram of stained nuclei. d) Out of the single nuclei population, intensity of SATB2 and BCL11B fluorescence are plotted. The negative population runs as a diagonal line. The positive populations are separated to the right or left of the diagonal line.

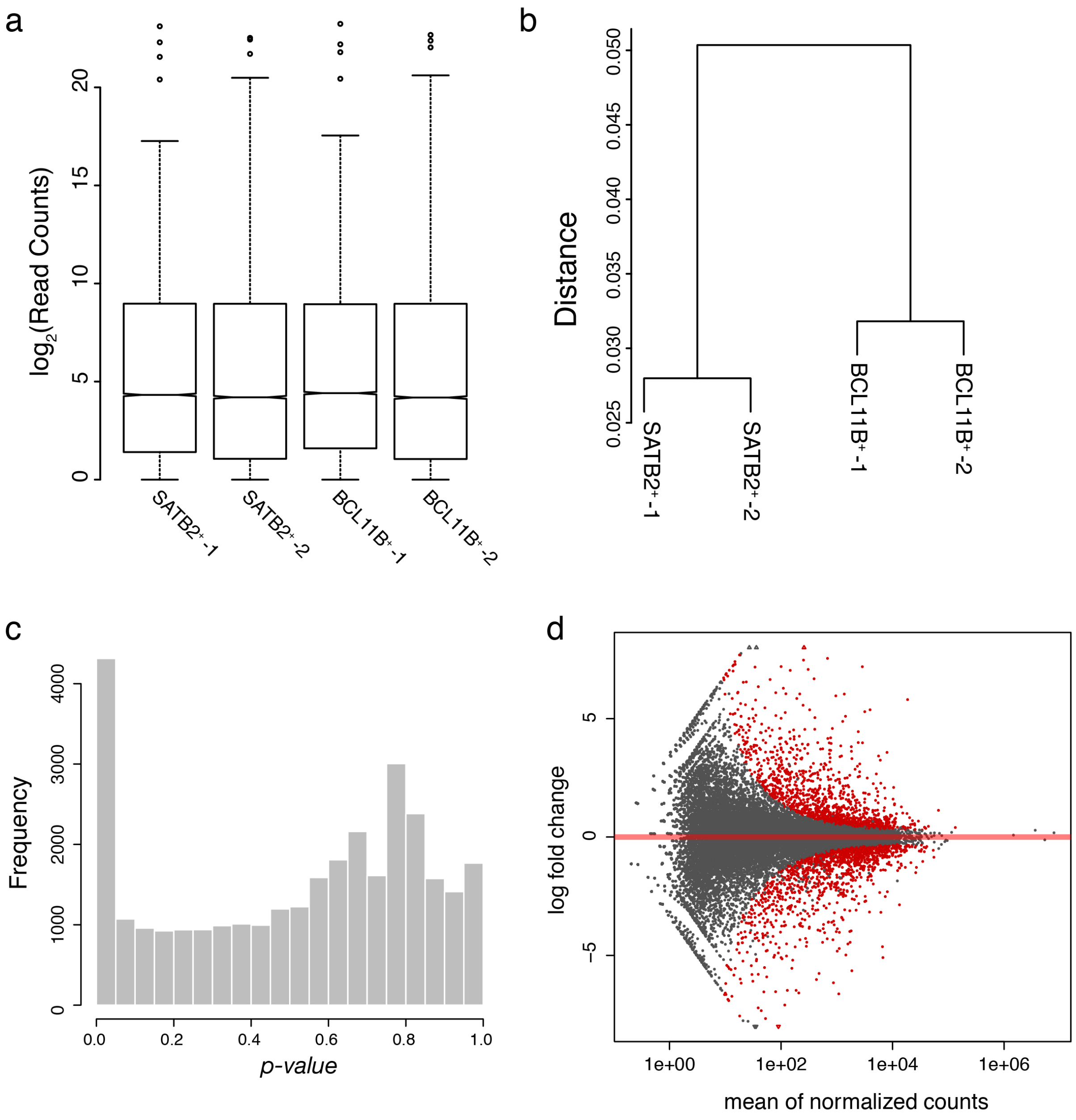

Supplementary Figure 2: Quality control for P30 mouse FIN-Seq data.

a) Log_2-transformed read distribution plot for sequenced mouse cortical samples. b) Dendrogram of read counts shows clustering of SATB2$^+$ samples and BCL11B$^+$ samples. c) A plot of frequencies of *p*-values shows an even distribution of null *p*-values. d) MA plot of log_2 fold change vs. mean of normalized counts. Red dots indicate genes that are differentially expressed.

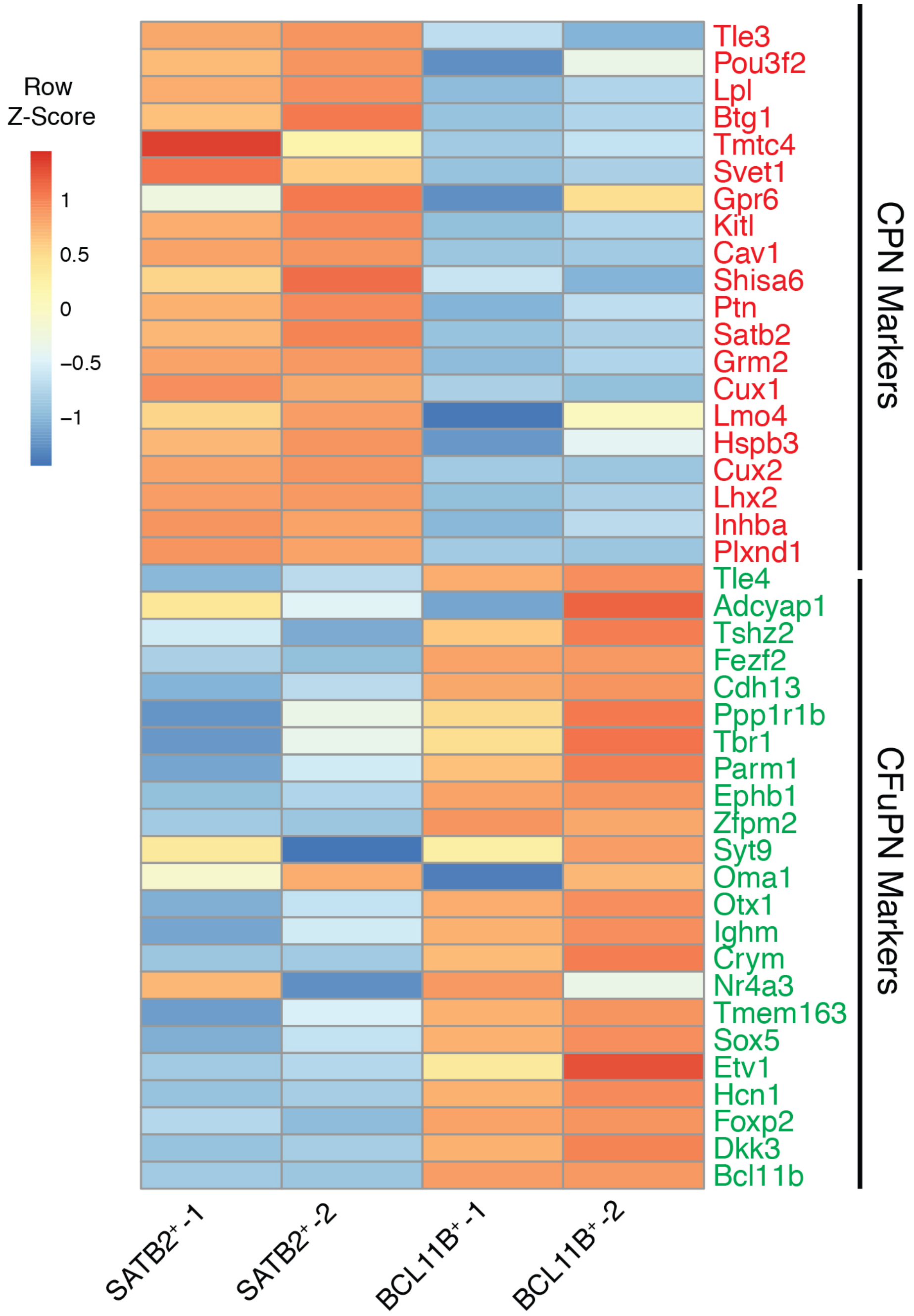

Supplementary Figure 3: Heatmap of known CPN and CFuPN markers.

CPN markers (in red) were enriched in the SATB2^{+} population while CFuPN markers (in green) were enriched in the BCL11B^{+} population.

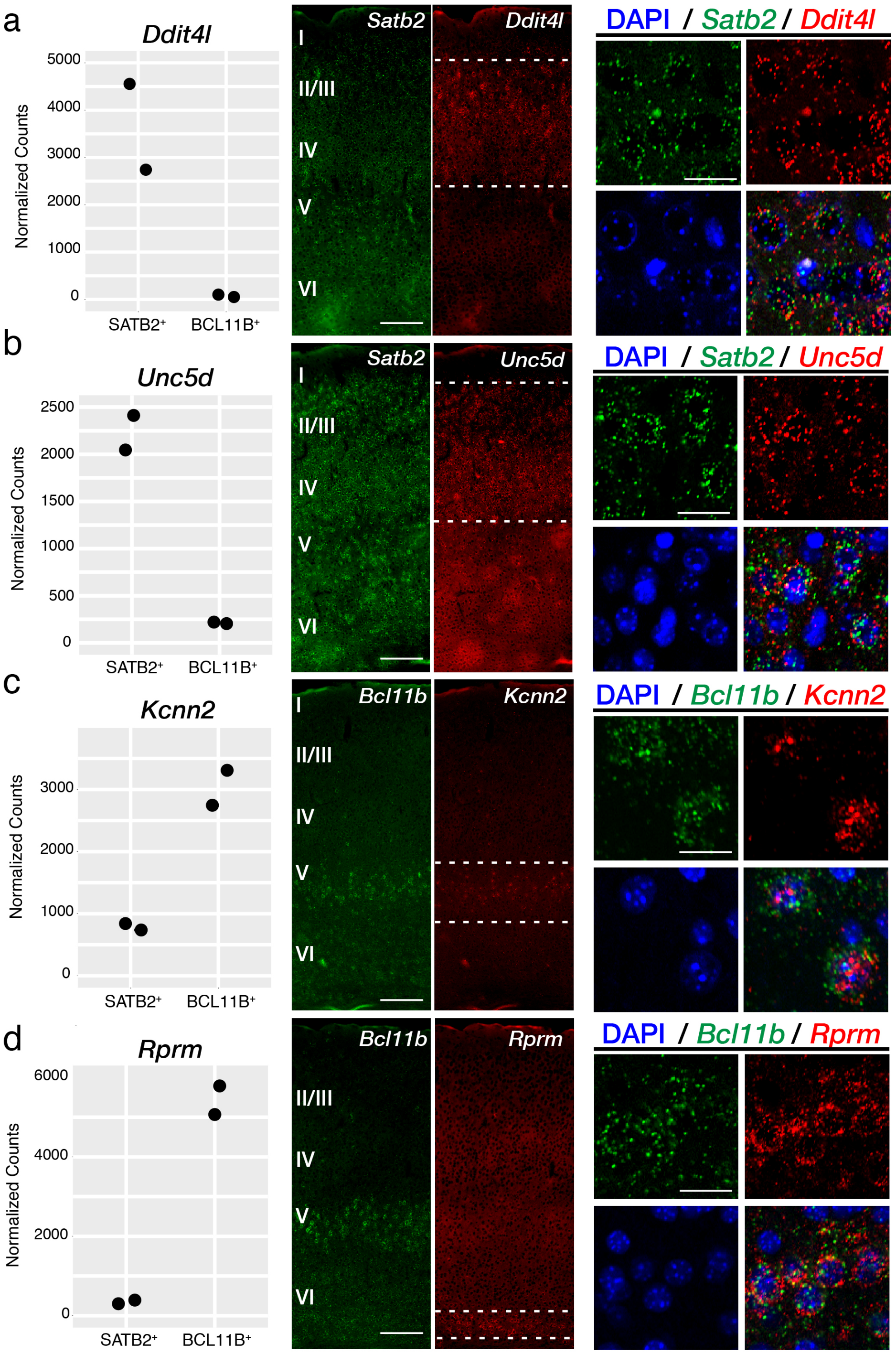

Supplementary Figure 4: Validation of subtype-specific transcripts by single molecule FISH.

Expression values from the RNA-seq are plotted (left panel). *Ddit4l* (a) and *Unc5d* (b) were expressed in the upper layers at P30 in the mouse neocortex (middle panel, between white dotted

lines). At this age, *Satb2* was expressed in subset of neurons of all layers (left panel). Merged higher magnification inset (right panel) shows expression of *Ddit4l* (a) and *Unc5d* (b) in *Satb2*$^{+}$ cells in layer 2/3. *Kcnn2* (c) and *Rprm* (d) were expressed in layer 5 and 6, respectively, in the mouse neocortex (middle panel, between white dotted lines). *Bcl11b* was expressed in neurons of layer 5 (left panel). Merged higher magnification inset (right panel) shows expression of *Kcnn2* (c) and *Rprm* (d) in *Bcl11b*$^{+}$ cells. Scale bars; 500 µm (a-d, middle panels), 20 µm (a-d, right panels).

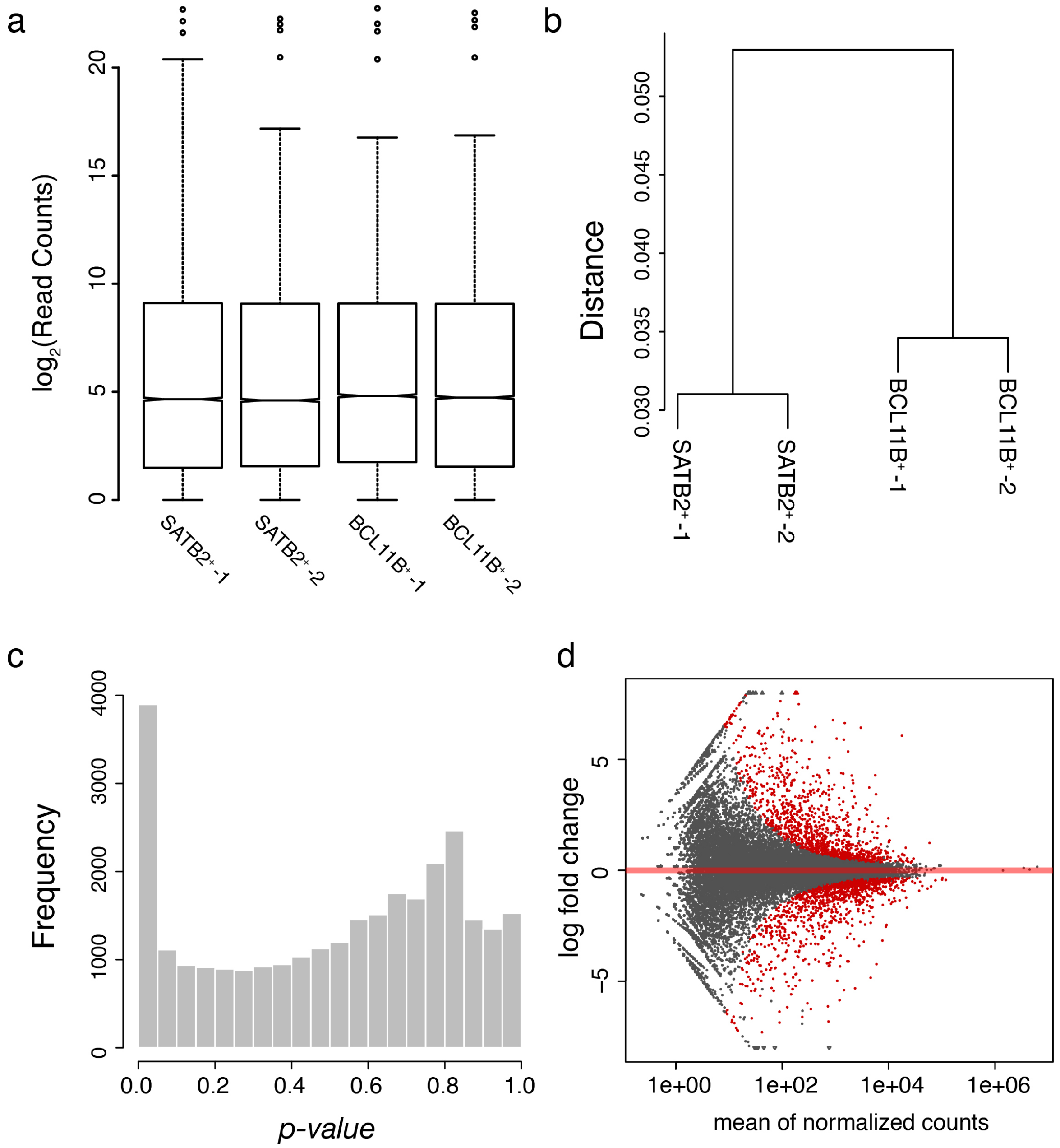

Supplementary Figure 5: Quality control for adult mouse FIN-Seq data.

a) Log_2-transformed read distribution plot for sequenced mouse cortical samples. b) Dendrogram of read counts shows clustering of $SATB2^+$ samples and $BCL11B^+$ samples. c) A plot of frequencies of *p*-values shows an even distribution of null *p*-values. d) MA plot of log_2 fold change vs. mean of normalized counts. Red dots indicate genes that are differentially expressed.

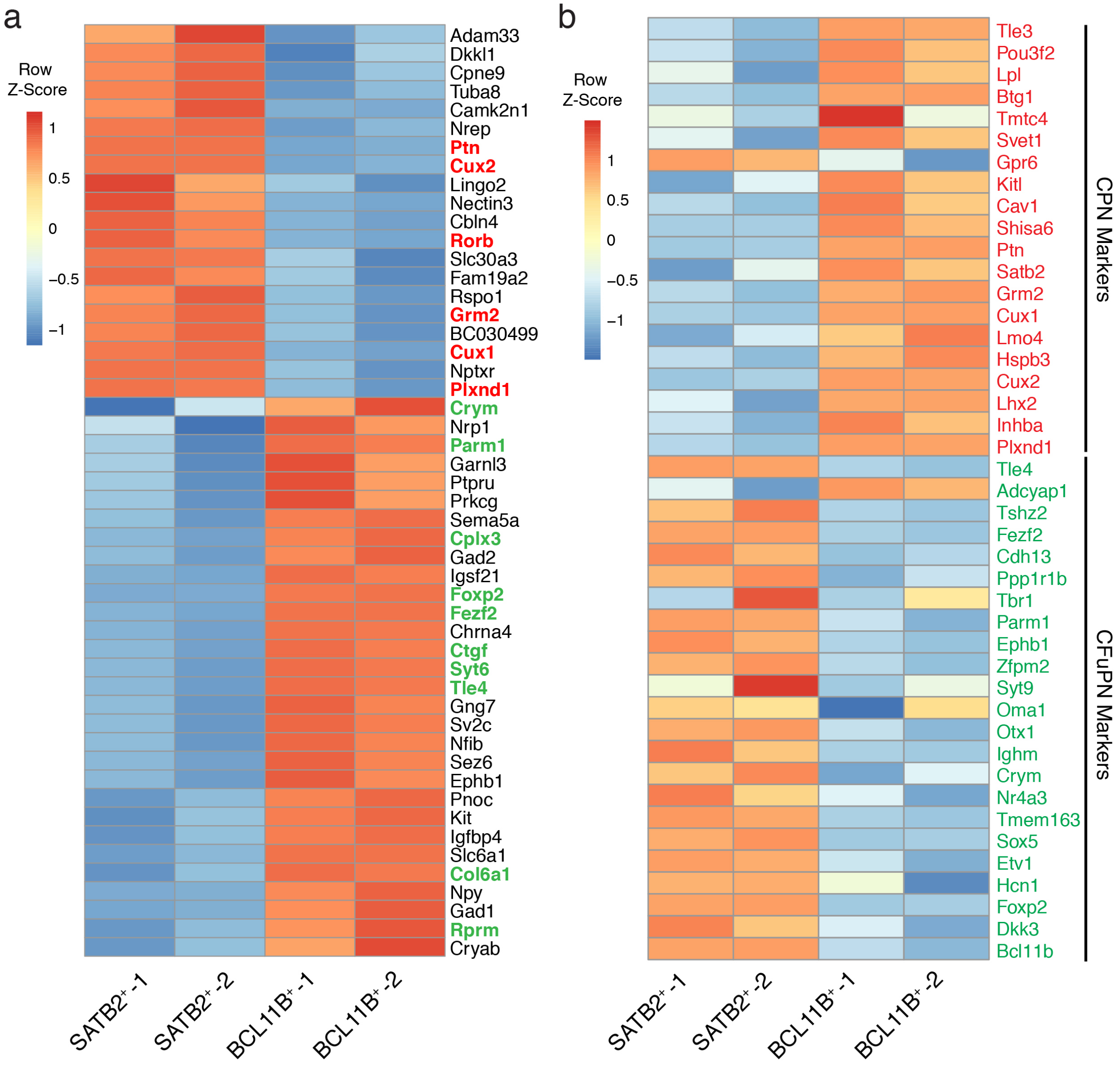

Supplementary Figure 6: Heatmap of unbiased top 50 genes and known CPN and CFuPN markers for the adult mouse FIN-Seq data.

a) Heatmap of unbiased top 50 differentially expressed genes between SATB2^{+} and BCL11B^{+} populations. Known markers of CPN (in red) were enriched in the SATB2^{+} population while known markers of CFuPN (in green) were enriched in the BCL11B^{+} population. b) CPN markers (in red) were enriched in the SATB2^{+} population while CFuPN markers (in green) were enriched in the BCL11B^{+} population.

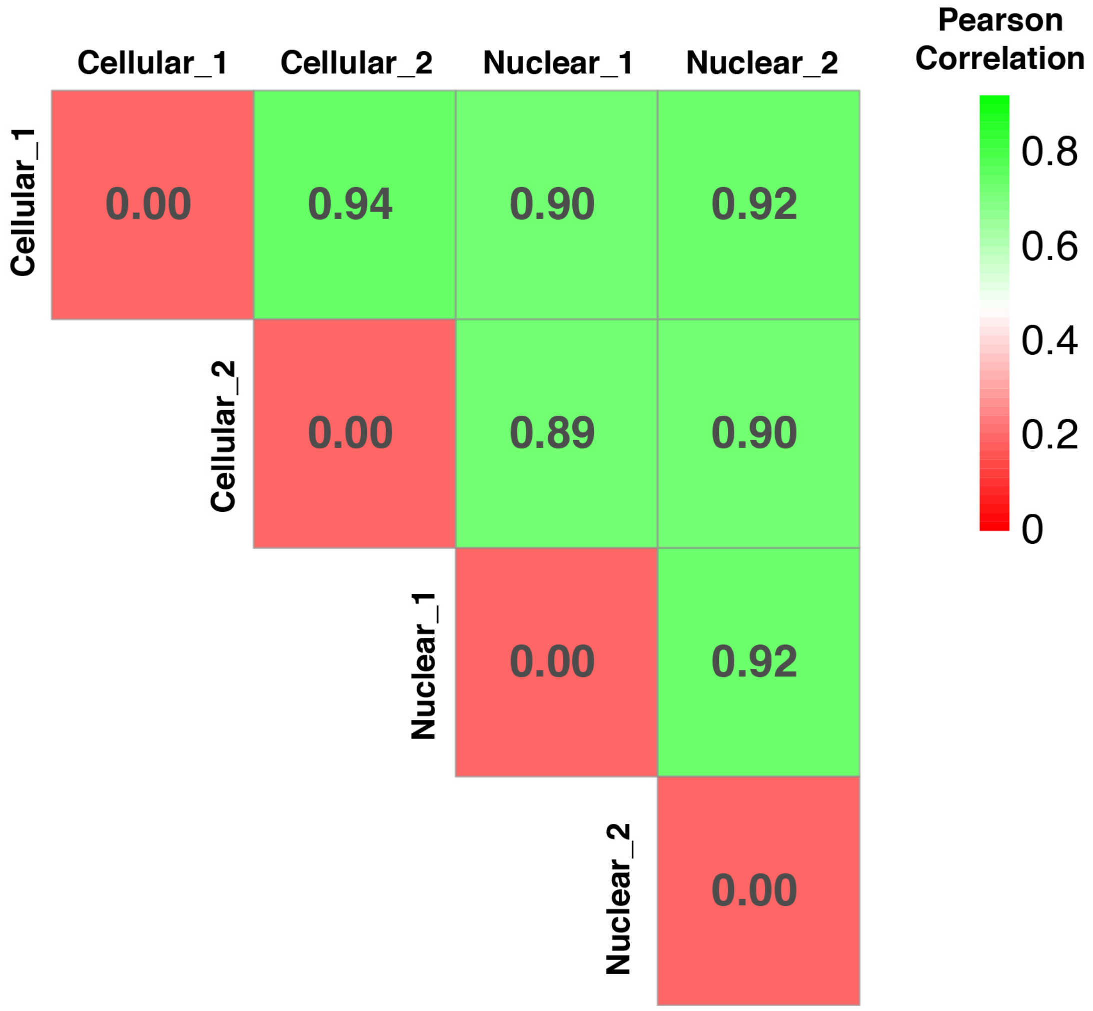

Supplementary Figure 7: Heatmap of correlation between cellular and nuclear transcriptomes.

Pearson correlation between cellular P7 BCL11B^{+} transcriptomes (n=2) and nuclear P7 BCL11B* transcriptomes (n=2) shows high correlation between cellular and nuclear transcriptomes.

a

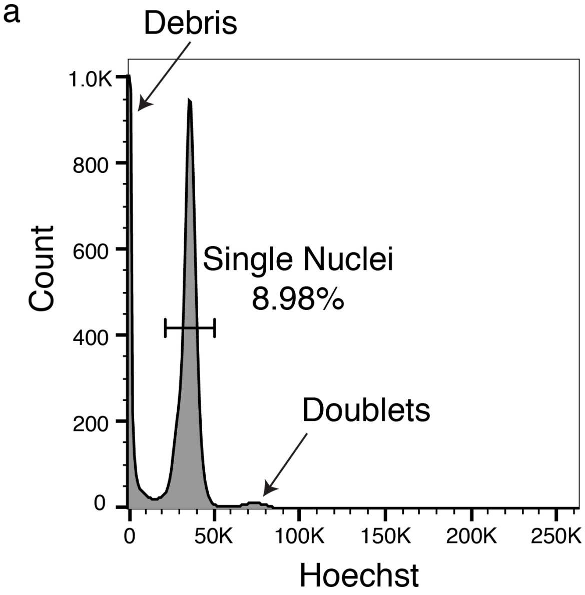

b

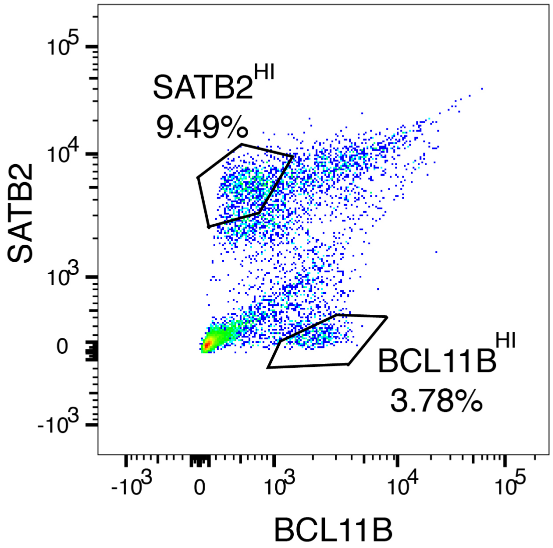

Supplementary Figure 8: FACS plot for obtaining SATB2^{HI} and BCL11B^{HI} populations.

a) All events are first plotted as a histogram of Hoechst intensity (405 nm laser). The peak near 0 is the debris that does not contain DNA. The second peak corresponds to single nuclei (8.98% of all events). The percentage of debris is higher in the human cortex. The peak to the right corresponds to doublets. b) Out of the single nuclei population, intensity of SATB2 and BCL11B fluorescence are plotted. The negative population runs as a diagonal line. The positive populations are separated to the right or left of the diagonal line.

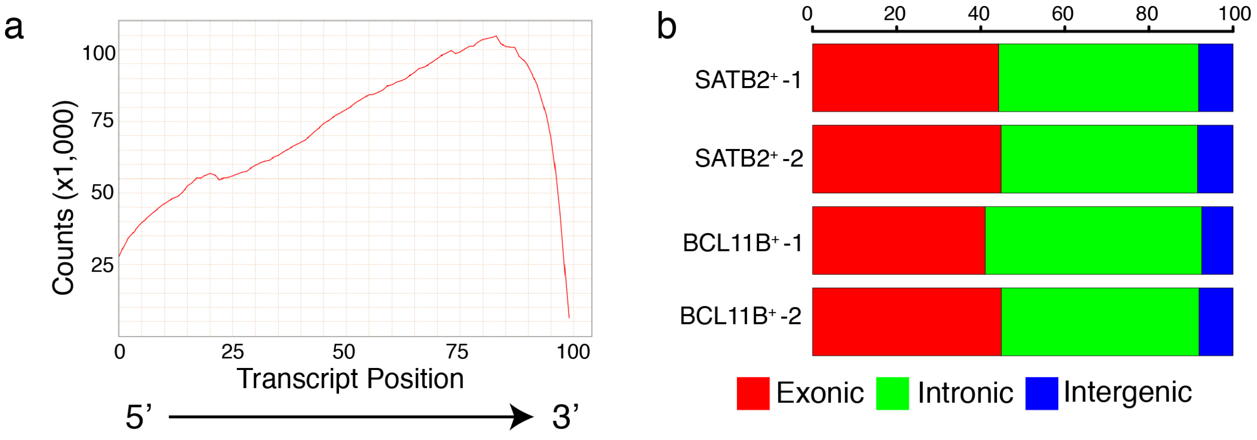

Supplementary Figure 9: Quality control for genome mapping of human cortical FIN-Seq reads.

a) Quantification of read counts mapped to transcript position (5' to 3') for every gene shows a strong 3' bias. b) Quantification of percentage of read counts mapped to exonic, intronic, or intergenic regions of the genome shows high percentage of intronic (green) reads.

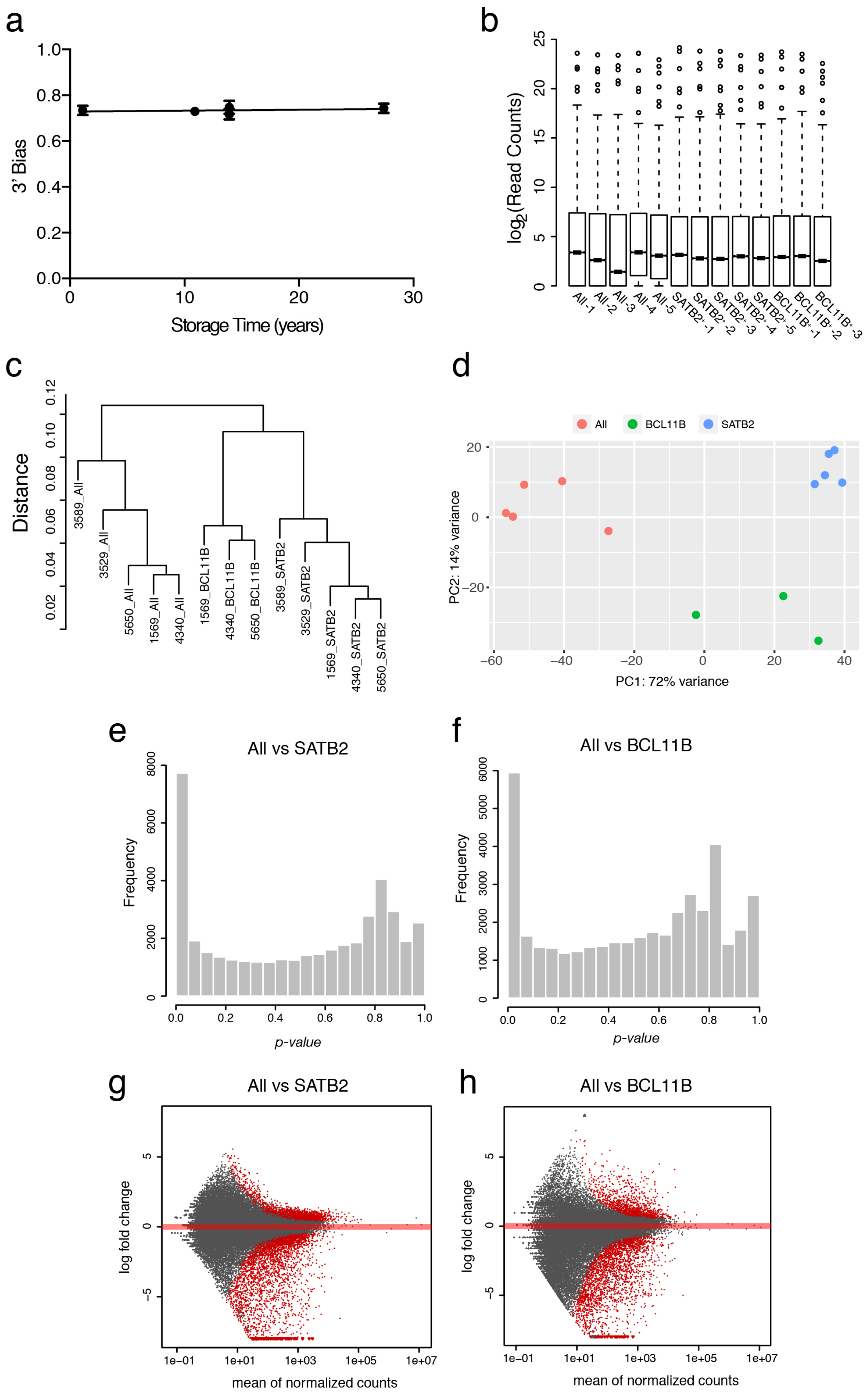
a
3' Bias
Storage Time (years)
b
log2(Read Counts)
All-1
All-2
All-3
All-4
All-5
SATB2+-1
SATB2+-2
SATB2+-3
SATB2+-4
SATB2+-5
BCL11B+-1
BCL11B+-2
BCL11B+-3
c
Distance
3589_All
3529_All
5650_All
1569_All
4340_All
1569_BCL11B
4340_BCL11B
5650_BCL11B
3589_SATB2
3529_SATB2
1569_SATB2
4340_SATB2
5650_SATB2
d
All
BCL11B
SATB2
PC2: 14% variance
PC1: 72% variance
e
All vs SATB2
Frequency
p-value
f
All vs BCL11B
Frequency
p-value
g
All vs SATB2
log fold change
mean of normalized counts
h
All vs BCL11B
log fold change
mean of normalized counts

Supplementary Figure 10: Quality control for adult human cortical FIN-Seq data.

a) A scatter plot of 3' bias with respect to the number of years that the samples have been stored. Data points are mean ± SD. b) Log_2-transformed read distribution plot for sequenced human cortical samples. c) Dendrogram of read counts shows clustering of $SATB2^+$ samples, $BCL11B^+$ samples, and All samples. d) PCA plot shows clustering of $SATB2^+$ samples, $BCL11B^+$ samples, and All samples. e-f) A plot of frequencies of *p*-values shows an even distribution of null *p*-values for the All vs. $SATB2^+$ population (e) or All vs. $BCL11B^+$ population (f) comparisons. g-h) MA plot of log_2 fold change vs. mean of normalized counts for the All vs. $SATB2^+$ population (g) or All vs. $BCL11B^+$ population (h) comparisons. Red dots indicate genes that are differentially expressed.

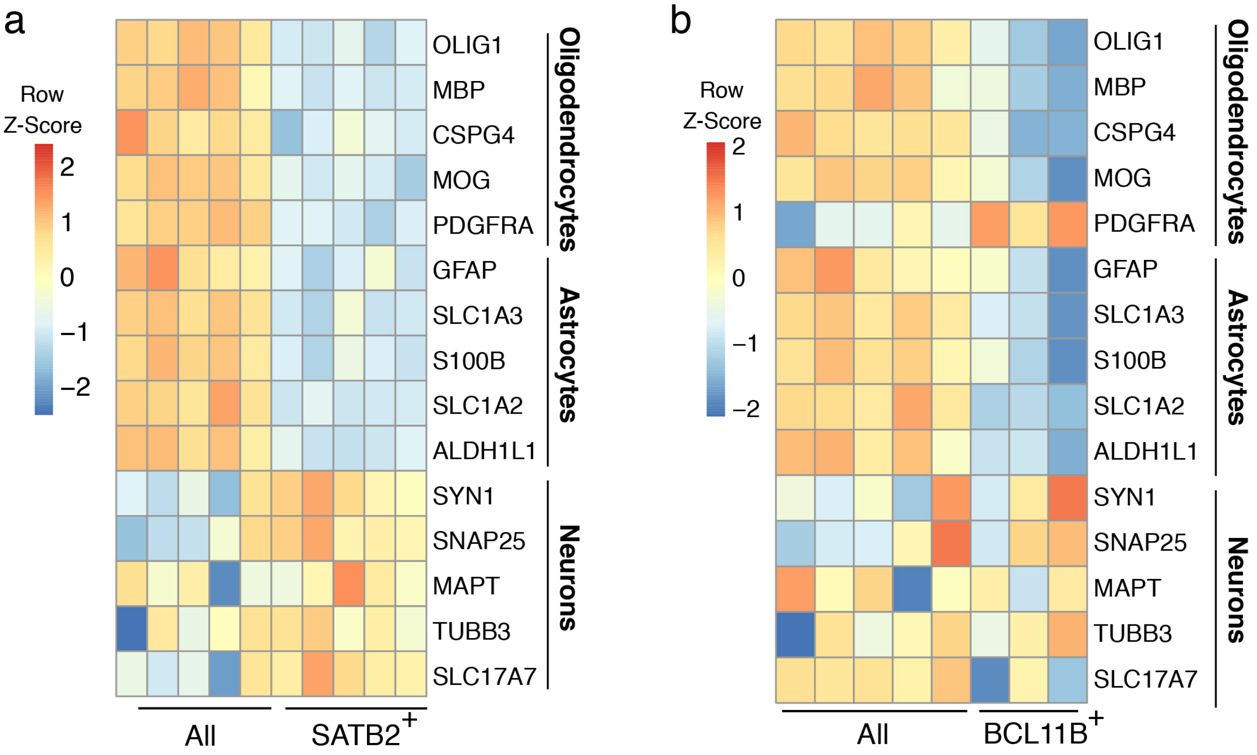

Supplementary Figure 11: BCL11B^{+} population contains specific inhibitory neuronal subtypes.

A heatmap representing relative expression levels of cell class markers in All and SATB2^{+} (a) or All and BCL11B^{+} (b) populations. Expression profile of five oligodendrocyte markers (OLIG1, MBP, CSPG4, MOG, PDGFRA), five astrocyte markers (GFAP, SLC1A3, S100B, SLC1A2, ALDH1L1), and five neuronal markers (SYN1, SNAP25, MAPT, TUBB3, SLC17A7) shows enrichment of neuronal markers in both populations.

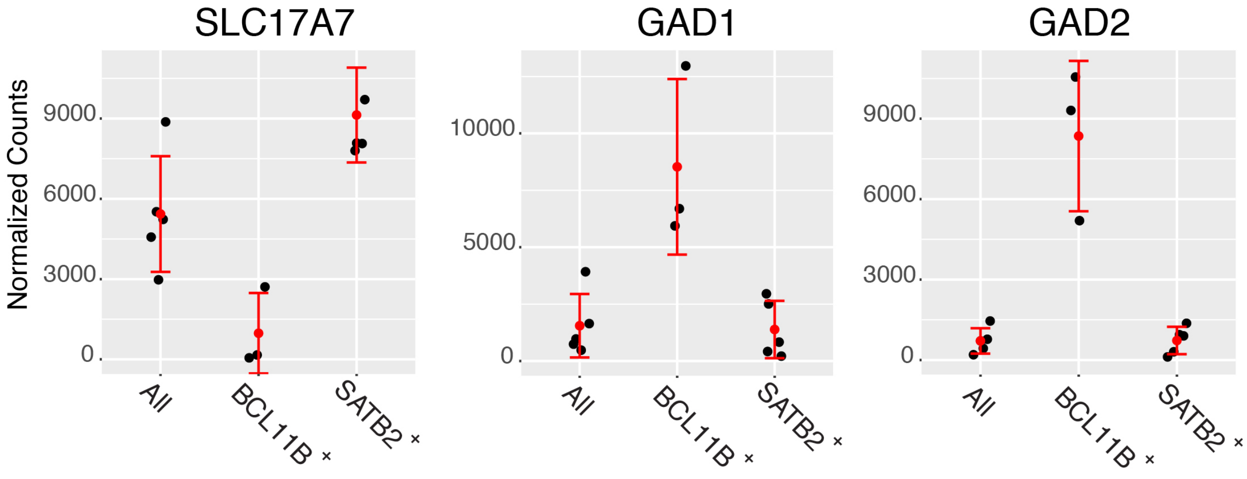

Supplementary Figure 12: SATB2$^+$ population contains mostly excitatory neurons while BCL11B$^+$ neurons also contain inhibitory neurons.

SLC17A7, an excitatory neuron marker, was enriched in SATB2$^+$ neurons (left panel). GAD1 and GAD2, inhibitory neuron markers, were highly expressed in the BCL11B$^+$ neurons in the human cerebral cortex (middle and right panels).

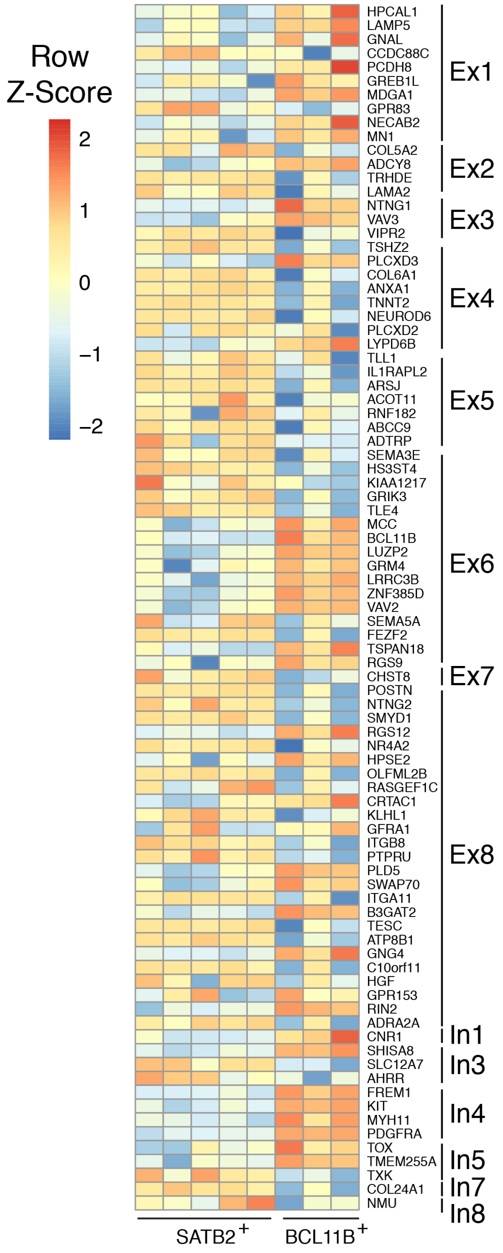

Supplementary Figure 13: Comparison of SATB2+ and BCL11B+ population shows some enrichment of Ex4 markers in the SATB2+ population and Ex6, In4, and In5 markers in the BCL11B+ population.

A heatmap representing relative expression levels of neuronal subtype markers previously identified by single nucleus RNA sequencing of a frozen human cortical sample.

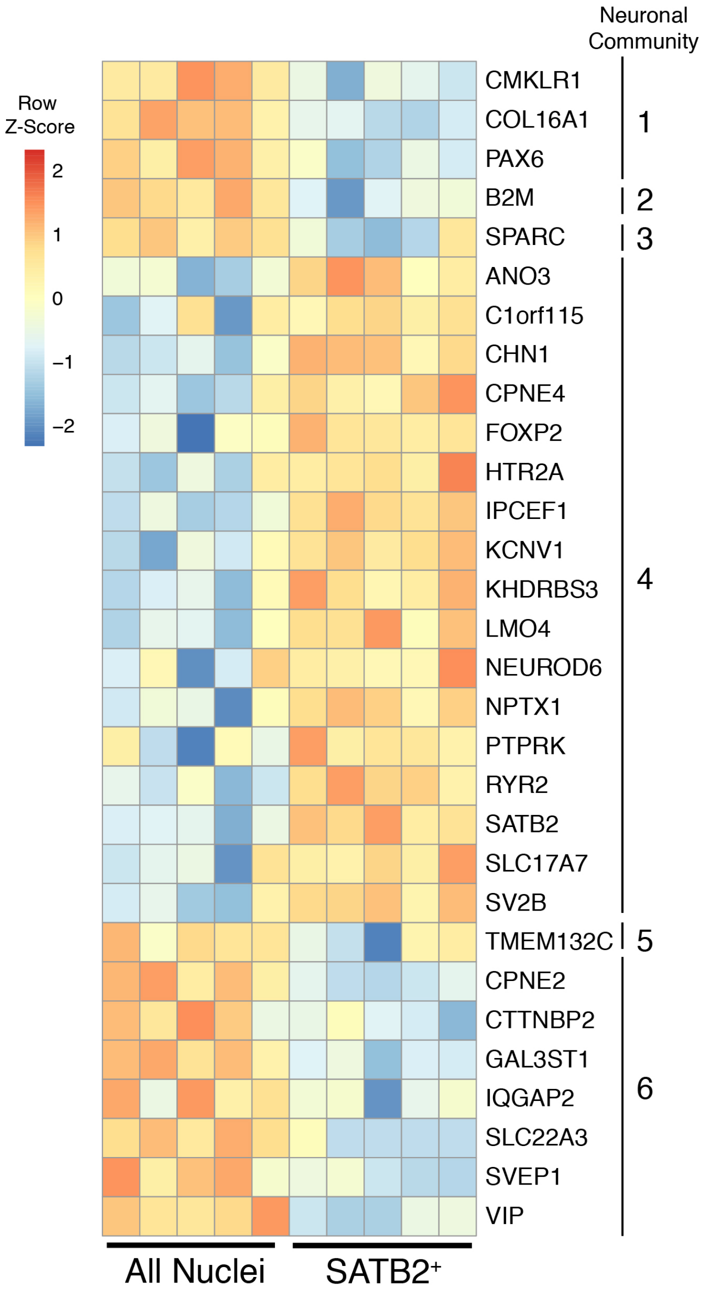

Supplementary Figure 14: SATB2$^+$ population represents neuronal community 4.

A heatmap representing relative expression levels of neuronal community markers previously identified by single cell RNA sequencing of a fresh human cortical sample. Markers of neuronal community 4, which expresses SATB2, were enriched in the SATB2$^+$ population.

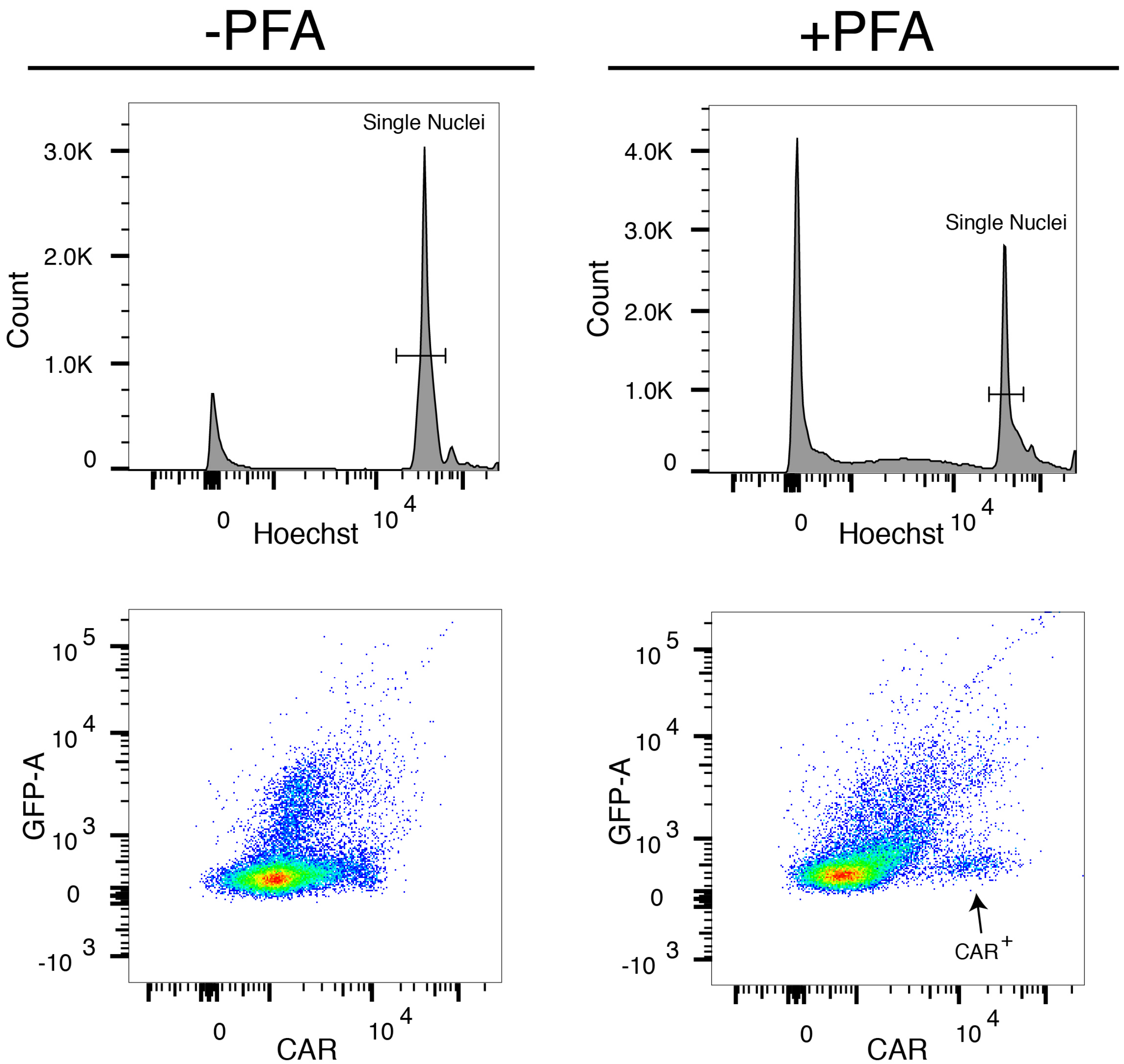

Supplementary Figure 15: Fixation is necessary for labeling cone photoreceptor nuclei with CAR.

FACS plots displaying histogram of Hoechst$^+$ nuclei with and without 4% PFA (upper panels: left panel without PFA, right panel with PFA). FACS plots displaying intensity of CAR staining on the x-axis and 488 nm autofluorescence on the y-axis (bottom panels). CAR$^+$ cluster was only visible with PFA (arrow).

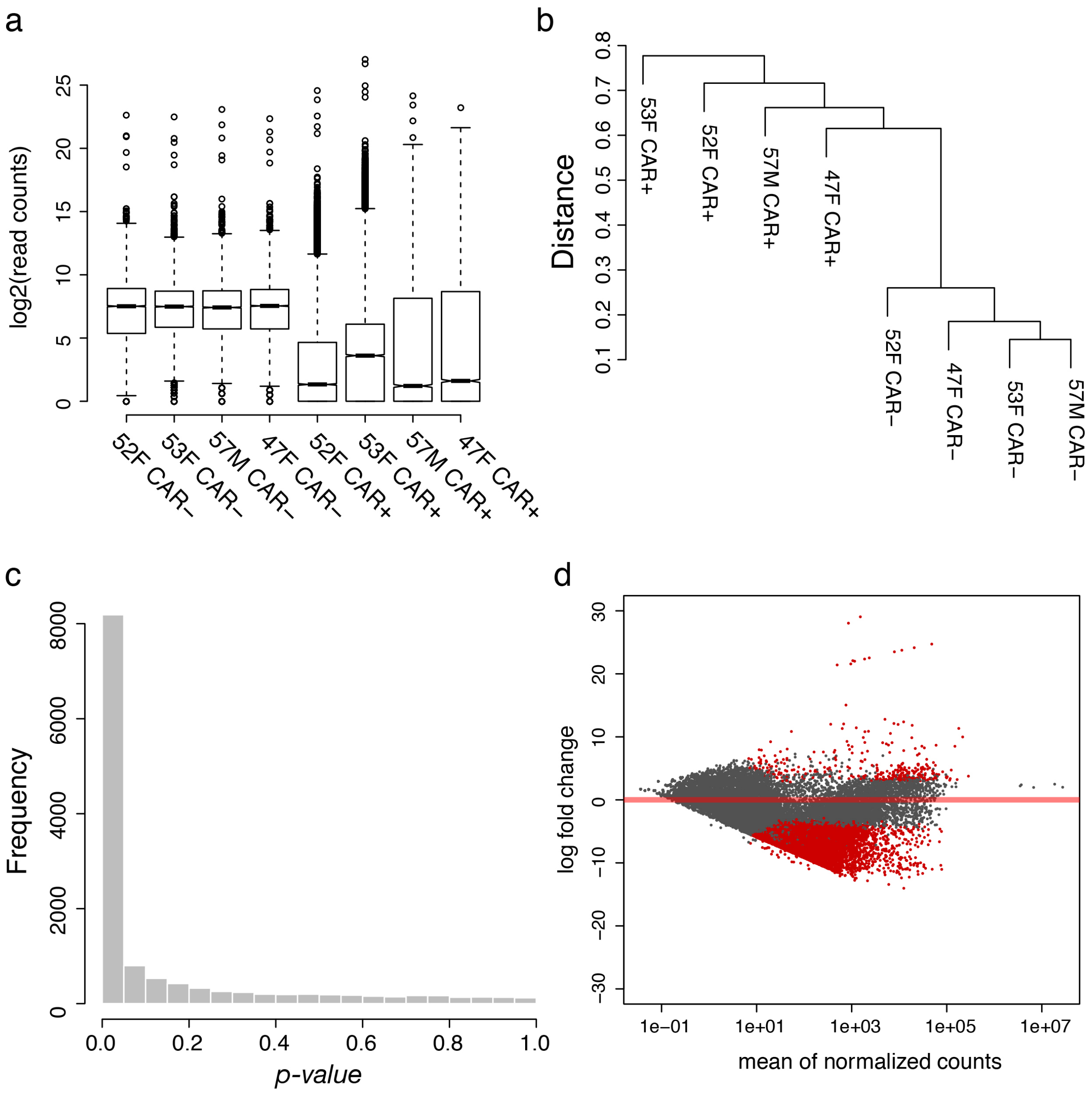

Supplementary Figure 16: Quality control for adult human retinal FIN-Seq data.

a) Log_2-transformed read distribution plot for sequenced human retinal samples. b) Dendrogram of read counts shows clustering of CAR^+ samples CAR^- samples. c) A plot of frequencies of *p*-values shows an even distribution of null *p*-values. d) MA plot of log_2 fold change vs. mean of normalized counts for the CAR^- vs. CAR^+ population comparison.

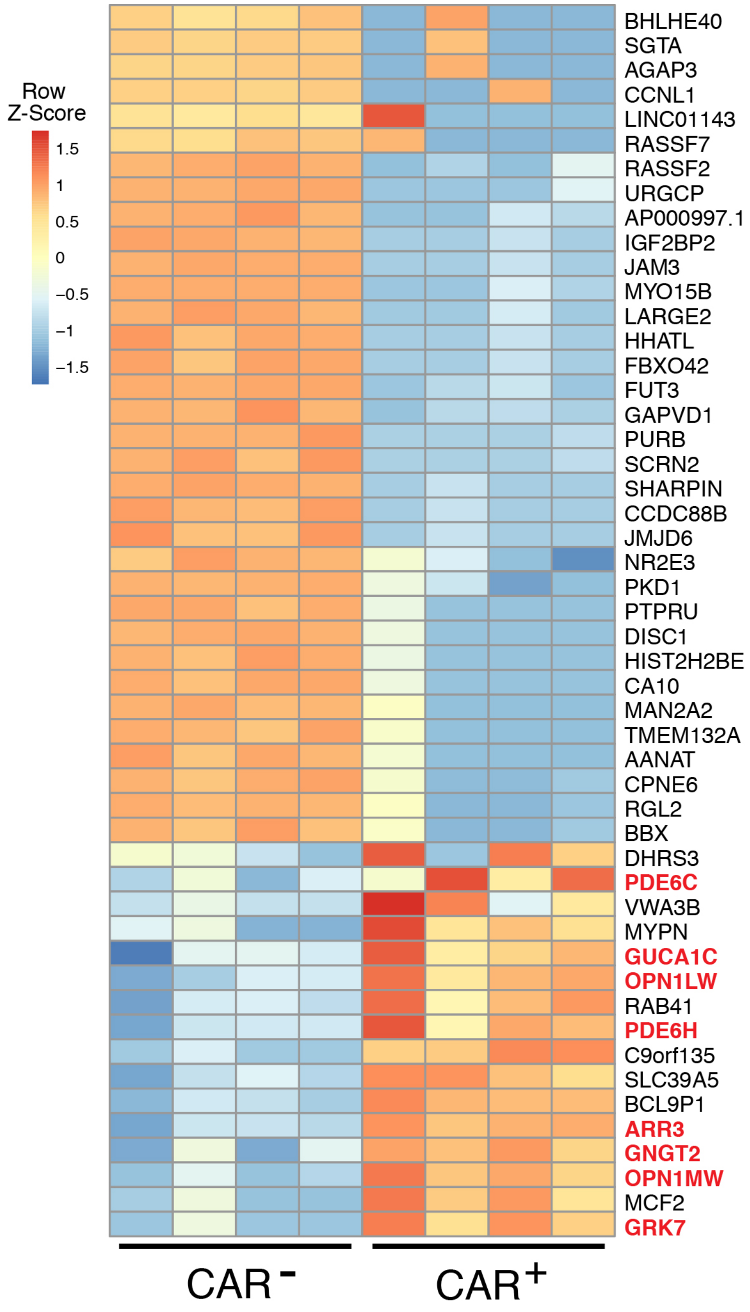

Supplementary Figure 17: CAR^{+} population contains mostly cone photoreceptors.

Heatmap of unbiased top 50 differentially expressed genes between CAR^{+} and CAR^{-} populations.

Known cone photoreceptor markers (in red) were enriched in the CAR^{+} population.
